# Phytoplankton dynamics in relation to seasonal variability and upwelling and relaxation patterns at the mouth of Ria de Aveiro (West Iberian Margin) over a four-year period

**DOI:** 10.1371/journal.pone.0177237

**Published:** 2017-05-04

**Authors:** Tânia Vidal, António José Calado, Maria Teresa Moita, Marina R. Cunha

**Affiliations:** 1 Departamento de Biologia & CESAM, Universidade de Aveiro, Campus Universitário de Santiago, Aveiro, Portugal; 2 IP Portuguese Inst Ocean & Atmosphere, IPMA, Lisbon, Portugal; 3 Departamento de Biologia & GeoBioTec, Universidade de Aveiro, Campus Universitário de Santiago, Aveiro, Portugal; 4 CCMAR, Universidade do Algarve, Campus de Gambelas, Faro, Portugal; University of Shiga Prefecture, JAPAN

## Abstract

From June 2004 to December 2007, samples were weekly collected at a fixed station located at the mouth of Ria de Aveiro (West Iberian Margin). We examined the seasonal and inter-annual fluctuations in composition and community structure of the phytoplankton in relation to the main environmental drivers and assessed the influence of the oceanographic regime, namely changes in frequency and intensity of upwelling events, over the dynamics of the phytoplankton assemblage. The samples were consistently handled and a final subset of 136 OTUs (taxa with relative abundance > 0.01%) was subsequently submitted to various multivariate analyses. The phytoplankton assemblage showed significant changes at all temporal scales but with an overriding importance of seasonality over longer- (inter-annual) or shorter-term fluctuations (upwelling-related). Sea-surface temperature, salinity and maximum upwelling index were retrieved as the main driver of seasonal change. Seasonal signal was most evident in the fluctuations of chlorophyll *a* concentration and in the high turnover from the winter to spring phytoplankton assemblage. The seasonal cycle of production and succession was disturbed by upwelling events known to disrupt thermal stratification and induce changes in the phytoplankton assemblage. Our results indicate that both the frequency and intensity of physical forcing were important drivers of such variability, but the outcome in terms of species composition was highly dependent on the available local pool of species and the timing of those events in relation to the seasonal cycle. We conclude that duration, frequency and intensity of upwelling events, which vary seasonally and inter-annually, are paramount for maintaining long-term phytoplankton diversity likely by allowing unstable coexistence and incorporating species turnover at different scales. Our results contribute to the understanding of the complex mechanisms of coastal phytoplankton dynamics in relation to changing physical forcing which is fundamental to improve predictability of future prospects under climate change.

## Introduction

Coastal upwelling occurs at localized regions of eastern ocean margins under the forcing of along-shore equatorward winds [1]. It involves the offshore displacement of usually nutrient-depleted surface waters and subsequent rise of cold and nutrient-rich deep waters into the coastal euphotic layer. These high nutrient pulses from deep waters are rapidly transformed in high amounts of biomass [2] and trigger phytoplankton succession [3,4]. They are then followed by relaxation events favoring the development of ‘blooms’ of different species [5,6] including potentially harmful algae [7]. By shaping the abundance, composition and structure of phytoplankton, upwelling events influence the functioning of marine ecosystems through overall productivity, nutrient cycling, and carbon export. Primary production ultimately supports food web dynamics and productive fisheries (e.g. [1,8–10]). There is recent evidence [11], that the global decrease in frequency and intensity of coastal upwelling events may have important socio-economic consequences both by the decline of fisheries and by increasing the frequency of harmful algal blooms (HAB).

The phytoplankton consist of a very large number of species in spatially and temporally dynamic assemblages. The availability of physical transport mechanisms (dispersal limitation), biological traits (growth rate, functional type, physical and chemical requisites) and biotic interactions (competition, predation) determine the local occurrence of varying subsets of the regional pool of species. In upwelling systems, different phytoplankton species use different mechanisms or functional strategies (e.g. mixotrophy) that allow them to take advantage of the multiple niches arising from the ever changing conditions in turbulence, temperature light and nutrient availability [12].

Upwelling favorable winds are usually seasonal, but pulse episodes as short as one day or extending for several weeks at a time [13] may occur all year-round. With a life-cycle timescale of days, phytoplankton responds rapidly to the physical disturbance and changing nutrient regimes induced by upwelling episodes even at the shorter scale [14]–the flux of nutrients regulates succession, while the frequency of water column destabilization is important for resetting the assemblage to early successional stages [3].

One of the major Eastern Boundary Upwelling Ecosystems but also one of the least studied is located along Iberia and Northwest Africa [1,15]. Upwelling along the Iberian Peninsula is relatively weak and seasonal, with summer maxima, although pulse episodes can occur at all seasons [1,16,17]. By their relevant contribution to total standing stock and primary production, these events have a significant impact on the food webs [18] supporting the productive fisheries and bivalve, shellfish and fish farming along the western coast of Portugal and Spain [19,20].

Over the last two decades bivalve and shellfish farming in Ria de Aveiro (NW Portugal) has been negatively impacted by the development of HAB and subsequently a phytoplankton monitoring programme has been established [20]. This programme relies on the examination of weekly samples taken from a fixed location at the mouth of Ria de Aveiro, one hour before the end of flood tide to ensure the predominance of coastal water entering the lagoon [21]. In the present study we examined four years’ weekly samples taken at this fixed station aiming the characterization of the phytoplankton assemblage and its dynamics in relation to the fluctuations of major environmental conditions. Taxonomically detailed information on the temporal fluctuations in composition and structure of the phytoplankton assemblage is provided with the following specific objectives: i) to interpret the seasonal and inter-annual variability of the assemblages in relation to the main environmental drivers; ii) to assess the effect of the oceanographic regime, namely changes in frequency and intensity of upwelling events, over the dynamics of the phytoplankton assemblage.

## Materials and methods

### Study area

The western coast of the Iberian Peninsula is under the influence of the northern component of the North Atlantic Upwelling region (reviewed by [22]). The Aveiro region is influenced by the subtropical branch of the Eastern North Atlantic Central Water (ENACWst) and by discharges from the coastal lagoon Ria de Aveiro, which acts as an important nutrient source especially during winter, much like what was described for the Tagus estuary at Lisbon Bay [23]. During the autumnal transition from upwelling to downwelling, apart from the onset of the poleward undercurrent on the slope, a relatively narrow poleward warm flow has been described on the inner shelf, inshore of a southward moving tongue of upwelled water responsible for transporting harmful dinoflagelates (and other phytoplankton communities) from northern Portuguese waters towards the Galician Rías Baixas [24]. Upwelling was identified as the major source of seasonal and spatial variability of phytoplankton along this segment of the Iberian Atlantic margin [25].

The Aveiro region is located in the middle section of western Iberia and features the largest bar-built coastal lagoon (inaccurately named a Ria) along the Atlantic coast of Portugal, extending 45 km N-S and about 10 km E-W [26]. Ria de Aveiro exchanges nearly 80% of its water mass with the ocean during each tidal cycle through a single outlet, whereas the freshwater input, from two main rivers and several smaller streams, is comparatively minor, except during periods of heavy rainfall [27]. The influence of coastal plankton is therefore strongly felt inside the lagoon during most of the year.

Phytoplankton productivity in the coastal area off Aveiro is affected by upwelling and commonly shows two maxima, a higher one in the spring and a lower one during autumn [28]. Several approaches to the development of models integrating phytoplankton dynamics and various environmental factors have been published for this area [29,30], although often with little knowledge of actual phytoplankton composition [31].

### Data collection

The locations sampled (within the Portuguese EEZ) are not privately-owned or protected in any way and the field studies did not involve endangered or protected species.

Samples were collected weekly from a single location at the mouth of Ria de Aveiro (Marégrafo; 40° 38' 38.90" N, 8° 44' 55.56" W), from June 2004 to December 2007. The samples were taken just below the surface, one hour before the end of flood tide to reduce the influence of the lagoon and obtain a good representation of coastal communities entering Ria de Aveiro. Water samples of 250 ml were immediately preserved with neutral formalin (final concentration formaldehyde: 2.4%) and stored in opaque plastic bottles. These samples were used for the quantitative analysis of phytoplankton species composition through the Utermöhl [32] counting technique.

Water temperature (°C) and salinity (practical salinity units, psu) were measured using a calibrated portable sensor WTW-LF 197, except between November 2006 and March 2007 when, due to malfunction of the device, measurements were taken separately by a hand thermometer and a refractometer. Differences in sensitivity and accuracy of the two methods resulted in minor irregularities in the measurements.

Two sets of three 250 ml replicates were collected for chlorophyll *a* determination: 1) one set for total photosynthetic biomass determination; 2) one set to estimate the nanoplanktonic (<20 μm, selected by filtration) fraction. The samples were filtered and the membrane filters were submitted to pigment quantification by fluorometric analysis with a Perkin-Helmer 204-A spectrofluorometer [33].

The daily upwelling index (Bakun index) was estimated following the method of Bakun [34], using wind speed and direction data measured every 6 h near Cape Carvoeiro by the Portuguese National Institute of Meteorology. The mean upwelling index was calculated as the average of the daily values from the week before each sample, as suggested by Bode and Varela [35].

### Phytoplankton counting

Subsamples of 25 ml were used for phytoplankton counting after sedimentation in a Kolkwitz chamber over 24 h or more. The counting was performed at 200x magnification (objective LD Plan-Neofluar 20x, NA 0.5) in phase contrast, with a Zeiss Axiovert 2000 inverted light microscope. Phytoplankton counts were expressed in cells per 100 ml. Organisms were identified to the lowest taxonomic level possible, herein designated by operational taxonomic units (OTUs). When organisms were too small (usually smaller than 20 μm) or otherwise impossible to identify at genus or species level, they were included in wider OTUs, often divided into size classes (listed in S1 Table). Very small-sized organisms (nano- or picoplankton), for which identification was impossible or inconsistent due to irregularities in fixation quality of this ever-present group, were excluded from the analyses; their contribution to the phytoplankton community is included in chlorophyll *a* concentrations measured for the filtrate fraction <20 μm.

### Data analysis

Data analyses were performed using the statistical package PRIMER 6 [36]. The main attributes of the phytoplankton assemblage structure were described by a set of four univariate descriptors: i) density, expressed as cells per 100 ml; ii) taxa richness (S), expressed as the number of operational taxonomic units (OTUs) whenever possible identified to species level; iii) diversity, expressed as the Shannon-Wiener index (H´; ln basis); iv) evenness, expressed as the Pielou’s index (J’) (Magurran 2004). Distributional analysis of the phytoplankton assemblage structure was carried out using k-dominance curves (cumulative abundances are plotted against species ranked by decreasing abundance contribution; [37]).

Changes in the composition of the phytoplankton assemblage were assessed by the turnover (e.g.[38]) between two consecutive periods according with season (e.g. spring and summer; seasonal turnover); and between consecutive changing in the upwelling conditions (e.g. downwelling after intense upwelling; short-term turnover). The index used was T = (L+G)/S; where L is the number of taxa lost, G is the number of taxa gained and S is the pooled number of taxa in the two consecutive periods; T values vary between 0 (no changes in the assemblage; G = 0 and L = 0) and 1 (complete renovation of the assemblage; L+G = S).

For the multivariate analysis the abundance data were first organised into a sample (185 samples collected) vs. OTU matrix. The OTUs with less than 0.01% of the total abundance in each of all the samples were discarded. Non-metric multidimensional scaling (MDS) ordination was performed using the Bray-Curtis similarity measure after logarithmic (log_10_ (n+1)) transformation of the data [39]. The data were analysed for the samples all together and for each of the four years separately. Analyses of similarities by randomisation/permutation tests (ANOSIM) were performed on the MDS results [40].

ANOSIM tests were directed to assess the significance of seasonal and interannual variability in the phytoplankton assemblage. The data from 2004 were excluded because this was not a complete sampling year. A two-way crossed layout was used with the following groups of samples: 2005, 2006 and 2007 for inter-annual variability, and winter (21 December to 20 March), spring (21 March to 21 June), summer (22 June to 21 September) and autumn (22 September to 20 December) for seasonal differences. This means that the tests for differences between “year” groups are averaged across all “seasonal” groups and vice versa [40]. Using the two-way crossed layout the effect of inter-annual variation can be assessed against a background of seasonally changing community structure. It is expected that the community will change seasonally and it is important to separate this effect from the inter-annual variability that might occur.

For the MDS plots of the four years analysed separately the significance of the seasonality was investigated by carrying out the RELATE–CYCLICITY routine [41]. This procedure calculates the Spearman correlation coefficient between the elements of two matrices of rank similarities, and then assesses the significance of the matching by a permutation procedure [42]. In this study, the first matrix was that of Bray-Curtis coefficients calculated for all pairs of samples. The second corresponded to a simple model matrix provided by the routine, with the sample relationships thought of as matching the inter-point distances of points placed equidistantly around a circle [36].

One-way ANOSIM tests were also performed to assess the significance of the changes in the oceanographic regime to the phytoplankton assemblage. The a priori groups of samples were defined as: intense upwelling (IU) associated with values of the Bakun upwelling index <-500 m^3^s^-1^km^-1^; weak upwelling (WU) associated with values <0 and >-500 m^3^s^-1^km^-1^, and downwelling (D) associated with positive values of the Bakun index (note that “intense” and “weak” are merely relative terms). The Bakun upwelling index values used were the average of the daily values during the week preceding each sample [35]. All samples were included in this analysis.

SIMPER routine (Similarity Percentages–species contributions) was applied to determine the percentage contributions of each OTU to the similarity within and dissimilarity between groups of samples both for the temporal and for the oceanographic regime analyses.

Finally, the correlations between the phytoplankton and environmental patterns was investigated using the RELATE routine [41]. This procedure calculates the Spearman correlation coefficient between the elements of two matrices of rank similarities, and then assesses the significance of the matching by a permutation procedure [42]. In this case, the first matrix was that of Bray-Curtis coefficients calculated for the biological data for all pairs of samples and the second corresponded to the Euclidean distances between samples calculated from the fourth-root transformed and normalised environmental data. The following environmental variables were considered: water temperature (°C), salinity (psu), chlorophyll *a* (total and <20 μm; mg.m^-3^), tidal range (m), upwelling index (average, maximum and minimum during the week before sampling; m^3^s^-1^km^-1^). The BEST–BIOENV procedure was used to identify the subsets of environmental variables that showed the best match with the patterns of organismal data. This is a stepwise procedure matching the Spearman correlation coefficients between the elements of the underlying similarity matrices [36].

## Results

### Environmental fluctuations

#### Temperature, salinity, chlorophyll a

**Fig 1:**
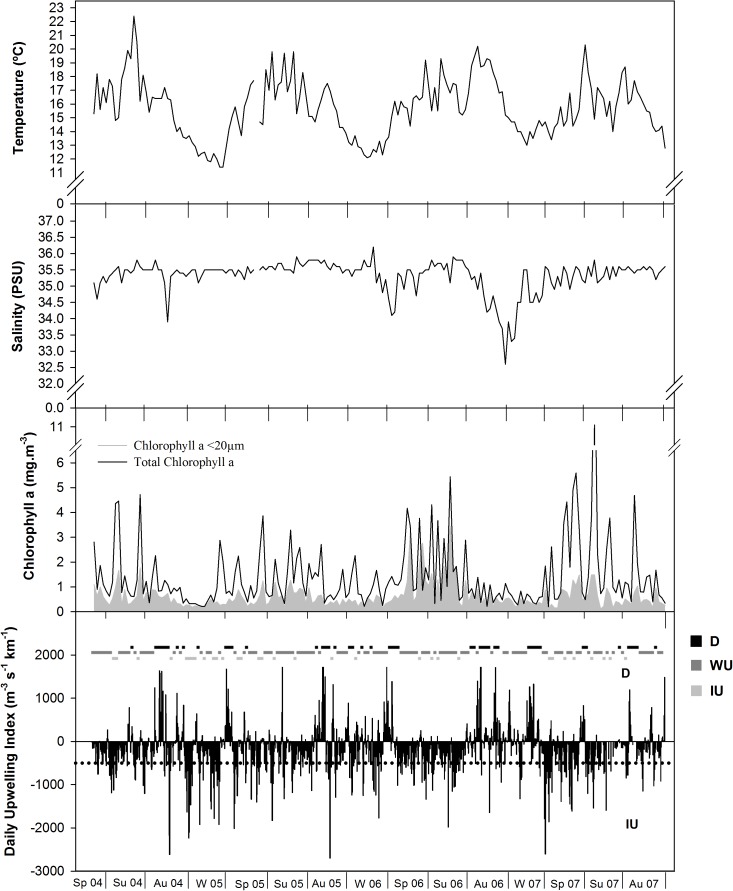
Fluctuations of the main environmental variables (temperature (°C); salinity (PSU); total chlorophyll *a* (mg.m^3^); chlorophyll *a* <20μm (mg.m^3^); daily upwelling index (m^3^.s^-1^.km^-1^) and upwelling bar- corresponding to the mean of the upwelling from the week before the sampling day throughout the study period (2004–2007). Sp: spring; Su: summer; A: autumn; W: winter. D: downwelling; WU: weak upwelling; IU: intense upwelling.

Water temperature ranged mostly between 13°C and 19°C, with an exceptionally warm peak of over 22°C in summer 2004 and a cold period with temperature below 12°C near the end of winter 2005. Temperature variation throughout the year was markedly seasonal, although variations of 3–4°C over a period of weeks were not uncommon, especially during spring and summer (Fig 1). Salinity variation was more limited and mostly around 35.5 psu, except during periods of heavy rainfall, which typically occurred during autumn and winter. During the study period, winter 2005 was unusually dry and salinity was hardly decreased, whereas in autumn 2006 and winter 2007 salinity decreased for several weeks down to a minimum just below 33 psu (Fig 1).

The chlorophyll *a* signal was also markedly seasonal with distinct oscillations mostly up to about 4–5 mg.m^-3^ during spring and summer. However, the overall chlorophyll *a* concentration also showed inter-annual variation: it was somewhat lower in 2005, a year during which the difference between spring, summer and autumn was also less marked; in contrast, higher concentrations of chlorophyll *a* were recorded in spring-summer 2007; the frequency of short-term variations in chlorophyll concentration was highest during spring-summer 2006, when the contribution of the phytoplankton fraction smaller than 20 μm was higher (Fig 1; note the different vertical scale between the two chlorophyll graphics).

#### Oceanographic regime

The fluctuations in the oceanographic regime varied seasonally and inter-annually during the study period (Fig 1). For the following description we considered the upwelling indices integrated weekly as described above (Material and Methods and represented graphically as a horizontal bar (Fig 1). A total of 85 successive periods were observed from which 23 under downwelling (D) conditions with an average duration of 2.1 weeks, 38 under weak upwelling (WU) with an average duration of 2.6 weeks and 24 under intense upwelling (IU) with an average duration of 1.2 weeks. Alternation between D and WU occurred more frequently during autumn and winter (73% of the cases) while alternation between IU and WU occurred mainly during the spring and summer (74% of the cases). Rapid change from D to IU, or vice versa, occurred in only nine occasions mostly during autumn or winter. The year 2006 was characterized by the alternation of rather long periods of WU and D with only four 1-week periods of IU alternating with WU during summer. In 2005 and 2007 the periods were shorter on average but, whereas in 2007 the change in the oceanographic regime was more gradual, in 2005 periods of IU were more frequent and rapid changes from D to IU, or vice versa, were often observed (five occasions).

### Phytoplankton assemblage

A total of 315 taxa were identified during this study and ascribed to five different Classes: 145 taxa of Bacillariophyceae, 141 of Dinophyceae, 15 of Haptophyta, two of Euglenophyceae and 12 of Chlorophyceae. After excluding the taxa with an average abundance contributing with less than 0.01% to the total, 136 taxa (76 Bacillariophyceae, 44 Dinophyceae, nine Haptophyta, two Euglenophyceae, five Chlorophyceae) were used for the multivariate analyses.

#### Inter-annual and seasonal variability

The MDS analysis shows that the samples from different years are largely overlapping (S1A Fig) but with a more obvious segregation of the seasonal groups (especially “winter” and “summer”; S1B Fig). The stress values are rather high (0.24) but not uncommon in the analysis of a large number of highly variable samples. More importantly, the ANOSIM tests indicate that both inter-annual and seasonal differences are highly significant (Table 1). The slightly higher value of R for season in the global test may be indicative of the predominance of seasonal changes over inter-annual changes. For the pairwise tests the estimated R values were higher for inter-annual differences between 2005 and 2007 and for seasonal differences between winter and summer.

**Table 1:** Results of the two-way ANOSIM (global and pairwise tests) for the MDS performed with samples collected in 2005, 2006 and 2007 to assess interannual and seasonal differences. 2004 was excluded because it does not represent a complete sampling year. The statistic estimated for each permutation is significant when its value is greater than or equal to the sample statistic. Significance level is calculated as the percentage of significant statistics in the total number of permutations used. R: Sample statistic.

Global test	R	Statistic significance	Number of permutations	Significant statistics
Interannual	0.589	0.1% ***	999	0
Season	0.621	0.1% ***	999	0
Interannual Pairwise tests:				
2005–2006	0.551	0.1% ***	999	0
2005–2007	0.717	0.1% ***	999	0
2006–2007	0.543	0.1% ***	999	0
Season Pairwise tests:				
Winter-Spring	0.662	0.1% ***	999	0
Winter-Summer	0.829	0.1% ***	999	0
Winter-Autumn	0.624	0.1% ***	999	0
Spring- Summer	0.436	0.1% ***	999	0
Spring-Autumn	0.686	0.1% ***	999	0
Summer-Autumn	0.547	0.1% ***	999	0

***: highly significant values.

A cyclic signal was revealed when the multidimensional ordination of samples was performed for each year separately (not shown); as the year progressed the structure of the phytoplankton assemblages gradually returned to the one from the departure date. The rank correlation test results from the RELATE-CYCLICITY routine proved the statistical significance of the annual cycle (2004: *ρ* = 0.320; 2005: *ρ* = 0.590; 2006: *ρ* = 0.411; 2007:, *ρ* = 0.554; p<0.001 in all cases).

**Fig 2:**
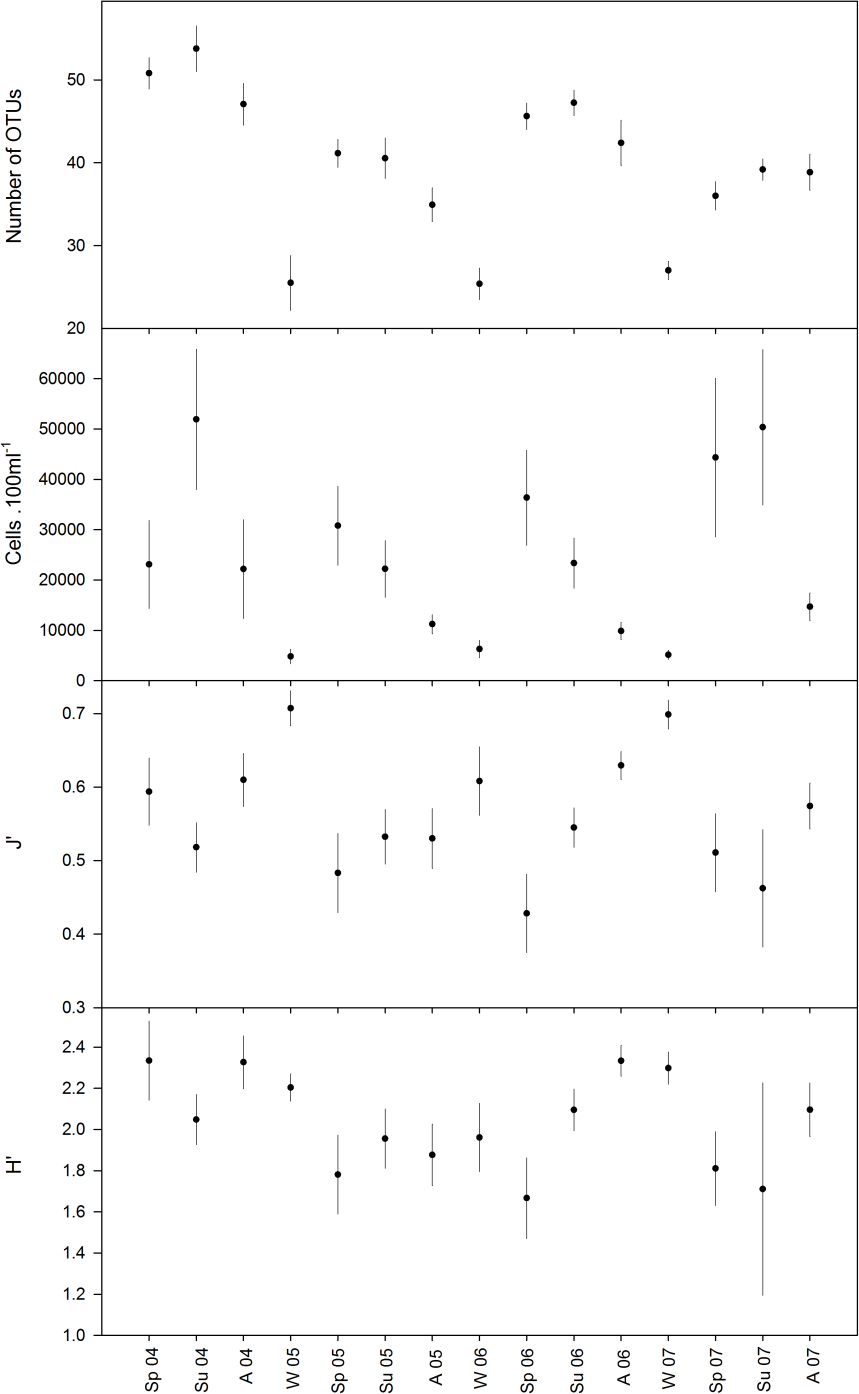
Community structure descriptors of the phytoplankton assemblage throughout the study period. Values shown are seasonal averages and respective standard error bars. Sp: spring; Su: summer; A: autumn; W: winter.

The temporal changes in the phytoplankton assemblages are clearly depicted by the variation in the community descriptors shown in Fig 2. Globally the winter assemblages showed the lowest taxa richness (seasonal average of 25 to 27 OTUs) and abundance (seasonal average of 4810 to 6283 cells per 100 ml) but relatively high diversity (seasonal average of H’: 1.96 to 2.30) and evenness values (seasonal average of J’: 0.6–0.7). During spring there was a dramatic increase in taxa richness (seasonal average of 36 to 51 OTUs) and abundance (seasonal average of 23093 to 44341 cells per 100 ml). In some years taxa richness was slightly increased in summer (2004, 2007) and even in autumn (2007); in general, high values were maintained from spring to autumn. Also, the increase in abundance continued moderately into summer of some years (e.g. 2004 and 2007) but tended to decrease to a minimal winter value. Spring and summer abundance values showed the highest variability (compare error bars in Fig 2). For abundance and taxa richness, inter-annual variability was expressed mainly by the variation in the period during which highest values were attained: summer in 2004, spring in 2005 and 2006 and autumn in 2007. The low values reached during winter showed very little inter-annual variability (Fig 2). The lowest diversity and evenness were found during spring (2005 and 2006) or summer (2004 and 2007) owing to the boost in abundance of a few dominant taxa. Because the observed inter-annual variability in these descriptors was high, the seasonal pattern is less clear than for taxa richness and abundance.

**Fig 3:**
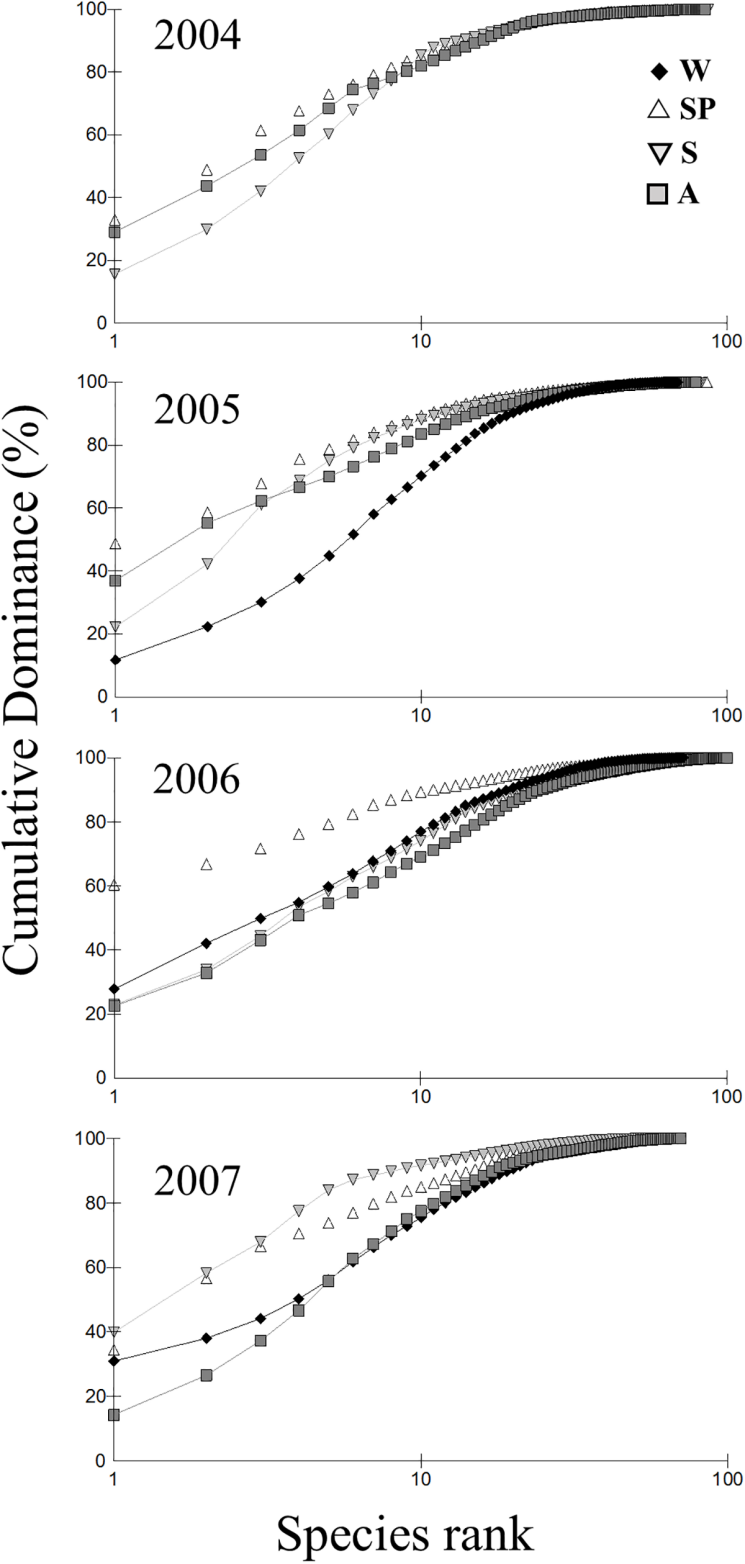
k-dominance curves for each season in the four years studied (2004–2007). Sp: spring; Su: summer; A: autumn; W: winter.

Inter-annual variability was more obviously depicted by the k-dominance curves (Fig 3 and S2 Table) showing different patterns of seasonal change in each year. In all years dramatic changes in community structure occurred from winter (low dominance) to spring (higher dominance) but in 2005 the spring structure was mostly maintained during the summer and autumn while in 2006 the spring assemblage attained the highest dominance (60% for the first taxa) but rapidly changed back to a low dominance structure (the summer and autumn curves are almost overlapping with winter). In 2007 there was a more gradual seasonal change with summer approaching the high dominance of spring, and autumn approaching the low dominance of winter.

**Fig 4:**
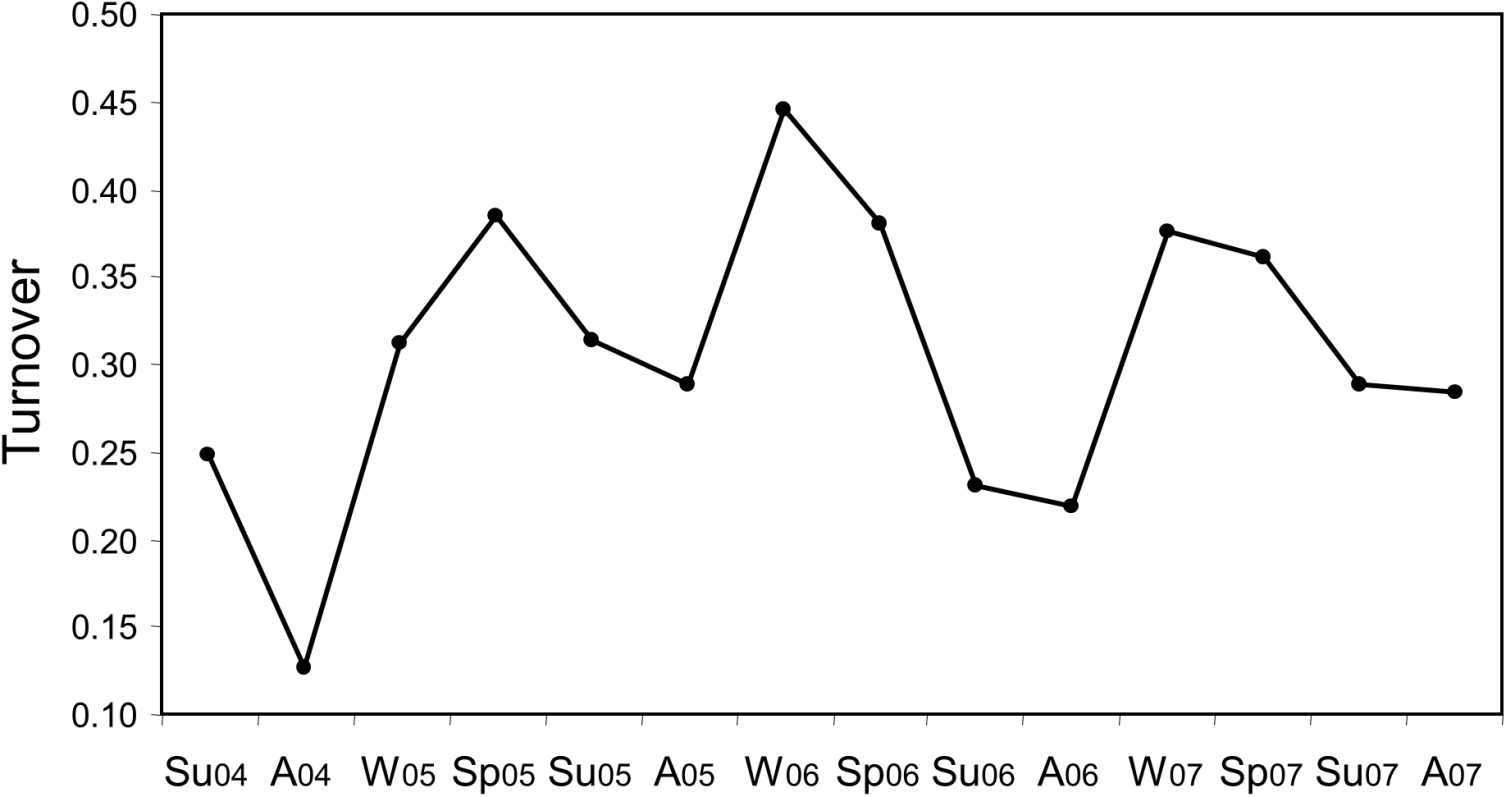
Turnover of the phytoplankton assemblage over the study period. Values shown represent changes in the taxonomic composition between two consecutive seasonal periods. Sp: winter to spring; Su: spring to summer; A: summer to autumn; W: autumn to winter.

Turnover values also showed a seasonal cyclicity in change (Fig 4), with the lowest values occurring invariably from summer to autumn (Fig 4: A) and the highest from autumn to winter (Fig 4: W) and winter to spring (Fig 4: Sp). The high turnover values were mainly due to the strong decrease in taxa richness in winter; these taxa then reappeared or were replaced by others during spring. During the study period the highest turnover values were observed in 2006 from the winter (Dec2005-Mar2006) to the spring (Mar-Jun 2006).

**Fig 5:**
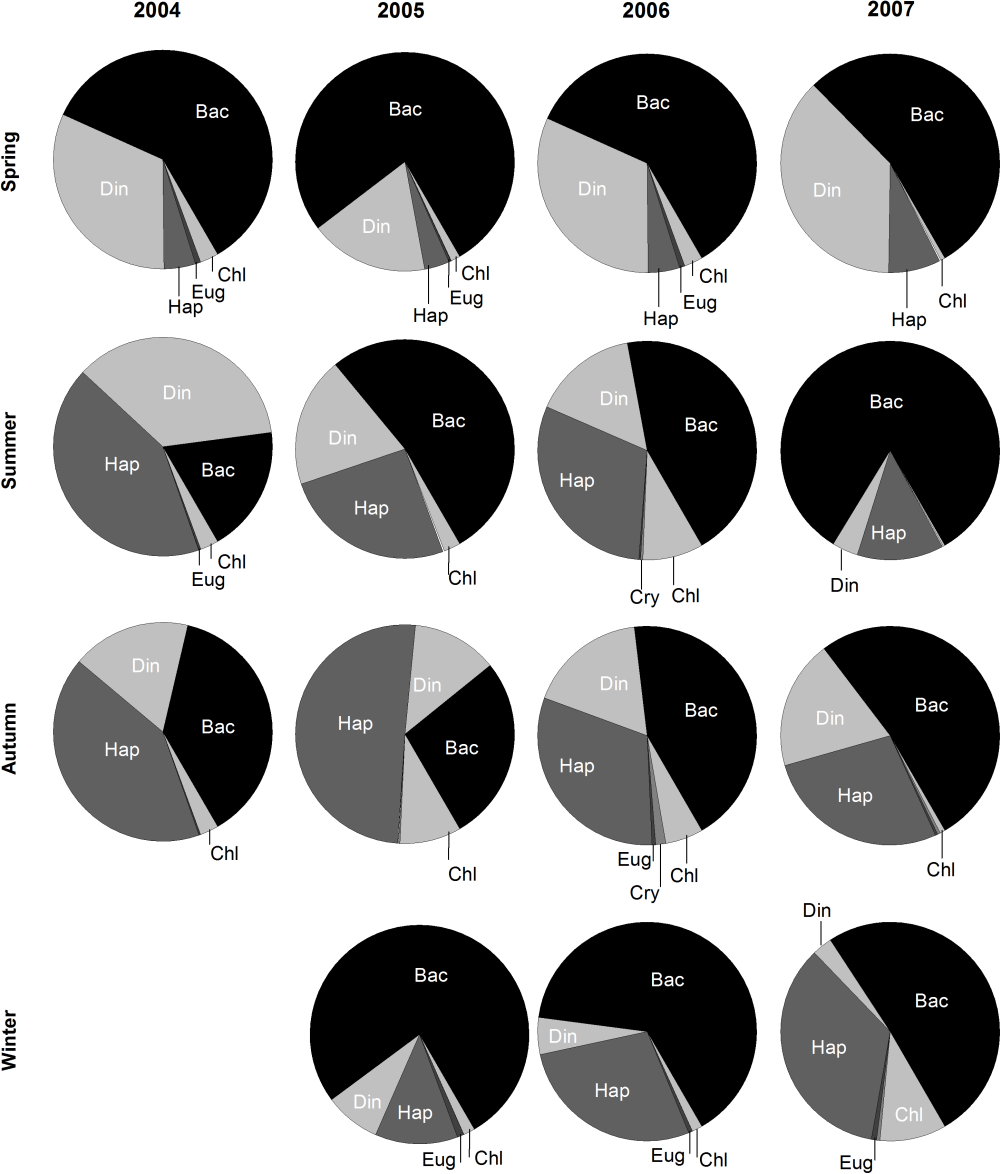
Relative contribution of the major taxonomic groups to the total abundance of the phytoplankton assemblages in each seasonal period of the four years studied. Bac: Bacillariophyceae; Din: Dinophyceae; Hap: Haptophyta; Eug: Euglenophyceae; Chl: Chlorophyceae.

With rare exceptions (in summer 2004 and autumn 2005) the phytoplankton assemblage at a higher taxonomic level (Fig 5) was dominated by Bacillariophyceae (diatoms) especially during spring and winter, Dinophyceae was the second most abundant group during spring, but was then replaced at this rank by haptophytes in the remaining seasons.

Two species had special relevance for the seasonal changes: the diatom *Leptocylindrus danicus*, which became highly dominant (up to 60% of the total abundance) during spring and usually maintained the first rank during summer, and the haptophyte *Emiliania huxleyi*, which was almost invariably among the dominant species and occasionally replaced *L*. *danicus* in the first rank of dominance, especially during autumn and winter (S2 Table). The SIMPER results (S3 Table) show a more detailed list of the main taxa that contributed to the similarity within and the dissimilarity between seasonal groups. The average similarities within seasonal groups (across all years) are relatively low reflecting year-to-year changes in species composition. The most important contributors were the dominant species *E*. *huxlei* and *L*. *danicus*, four other Bacillariophyceae (*Cylindrotheca closterium*, *Paralia sulcata*, centric diatoms A and pennate diatoms A) and one Dinophyceae (*Scrippsiella* cf. *trochoidea*). Each of these OTUs contributed less than 10% to the average similarity within seasonal groups. The average dissimilarities between seasonal groups reached up to 65.8% (summer vs. winter) but were accounted for by a large number of taxa with very low individual percentage contributions (<3.5%). Again, the chain-forming diatom *L*. *danicus* was relevant by its drastic seasonal fluctuation and typically low values during winter. Other relevant OTUs were the large-sized *Chaetoceros* spp. A, *Pseudo-nitzschia* spp. A and *Prorocentrum minimum*, dominant during spring, *Pseudo-nitzschia* spp. C (small-sized), dominant during summer, and the small-sized centric diatoms B, dominant during autumn. Noteworthy is the occurrence of potentially harmful algae, namely *Dinophysis acuta*; the toxin production by this species even at low cellular density is a recurrent cause of shellfish poisoning outbreaks. *Dinophysis acuta* occurred at higher densities mainly during summer but also in autumn (S3 Table) and in 2005 was among the dominant species in these seasons (S2 Table).

#### Phytoplankton assemblage vs oceanographic regime

The MDS plot for all samples coded by oceanographic regime groups (S2 Fig) does not show a clear segregation of the three groups of samples (IU, WU, D). However, the global ANOSIM test indicates a significant effect of the oceanographic regime, and pairwise tests further confirm significant differences between the assemblages of weak and intense upwelling periods (WU and IU, respectively), and between the assemblages of intense upwelling and downwelling (D). No significant differences were found between the assemblages of weak upwelling and downwelling (Table 2).

**Table 2:** Results of the one-way ANOSIM (global and pairwise tests) for the MDS performed with all samples collected. Differences between samples collected during periods of intense upwelling (IU), weak upwelling (WU) and downwelling (D) were assessed. The statistic estimated for each permutation is significant when its value is greater than or equal to the sample statistic. Significance level is calculated as the percentage of significant statistics in the total number of permutations used. R: Sample statistic.

	R	Statistic significance	Number of permutations	Significant statistics
Global test:				
Upwelling	0.061	1.8% *	9999	180
Pairwise tests:				
WU-IU	0.080	4.7% *	9999	468
WU-D	0.040	9.5% ns	9999	952
IU-D	0.103	0.3% **	9999	31

*: significant;

**: very significant, ns: non significant values.

**Fig 6:**
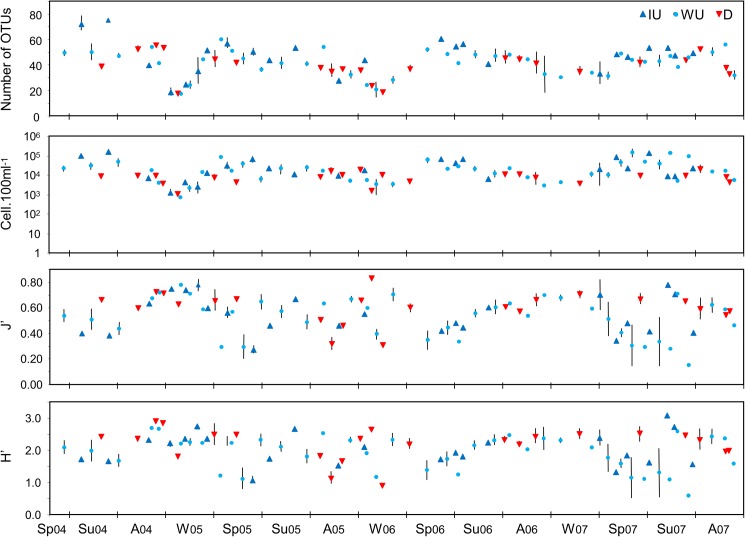
Community structure descriptors of the phytoplankton assemblage throughout the study period. Values shown are the averages and respective standard error bars for the different periods of upwelling conditions. IU: intense upwelling; WU: weak upwelling; D: downwelling.

The phytoplankton assemblage descriptors plotted according to the changes in the oceanographic regime are shown in Fig 6. The seasonal influence on the taxa richness and abundance appears to override the influence of the oceanographic regime, but considering that intense upwelling occurs predominantly during spring and summer and downwelling during autumn and winter the two factors are expected to be closely inter-related. Note that differences between seasonal averages of samples under the same physical forcing regime are smaller (small error bars) in winter of all years, and also throughout the year of 2006 (more stable oceanographic conditions).

The average number of taxa and density of the phytoplankton assemblage under intense upwelling conditions are higher than those observed under downwelling conditions (IU vs D, n = 31 and 47, respectively, average ± standard error across seasons: 46.3 ± 2.8 vs 41.4 ± 1.5 OTUs, post-hoc comparison: p>0.05, ns; 38996 ± 8061 vs 9466 ± 1050 cells.100 ml^-1^, p<0.001). However, the differences observed in the density and especially in the taxa richness (difference non significant between IU and D) are less marked that the ones observed between seasons (summer vs winter, n = 50 and 38, respectively: 47.8 ± 1.5 vs 30.2 ± 1.6 OTUs, p<0.001; 35641 ± 5471 vs 5514±801 cells.100 ml^-1^, p<0.001). More interestingly, the fluctuations of the oceanographic regime appear to have a stronger influence on the diversity and evenness (H’ and J’). The more intense (more periods of IU) and more frequent changes (shorter periods) in 2005 and 2007 appear to severely disrupt the seasonal pattern in the variation of diversity (H’) and evenness (J’) as opposed to 2006 (longer periods of WU few periods of IU) when the seasonal pattern was maintained (Fig 6). On average, the highest diversity and evenness were reached during D periods and the lowest during WU (D vs WU, n = 47 and 107, respectively: H’ = 2.24±0.07 vs 1.93±0.06, p<0.01; J’ = 0.607±0.018 vs 0.526±0.016, p<0.01). Also, the occurrence of frequent short events of IU during the spring and summer of 2007 led to large fluctuations in chlorophyll a concentrations that peaked at 11 mg.m^-3^. However, a similar pattern of IU events during the winter of 2005 had no such effect (Fig 1). Under more stable conditions (mostly WU) during the spring and summer of 2006, the increases in chlorophyll a concentrations were mostly contributed by small-sized species (<20μm, Fig 1).

**Fig 7:**
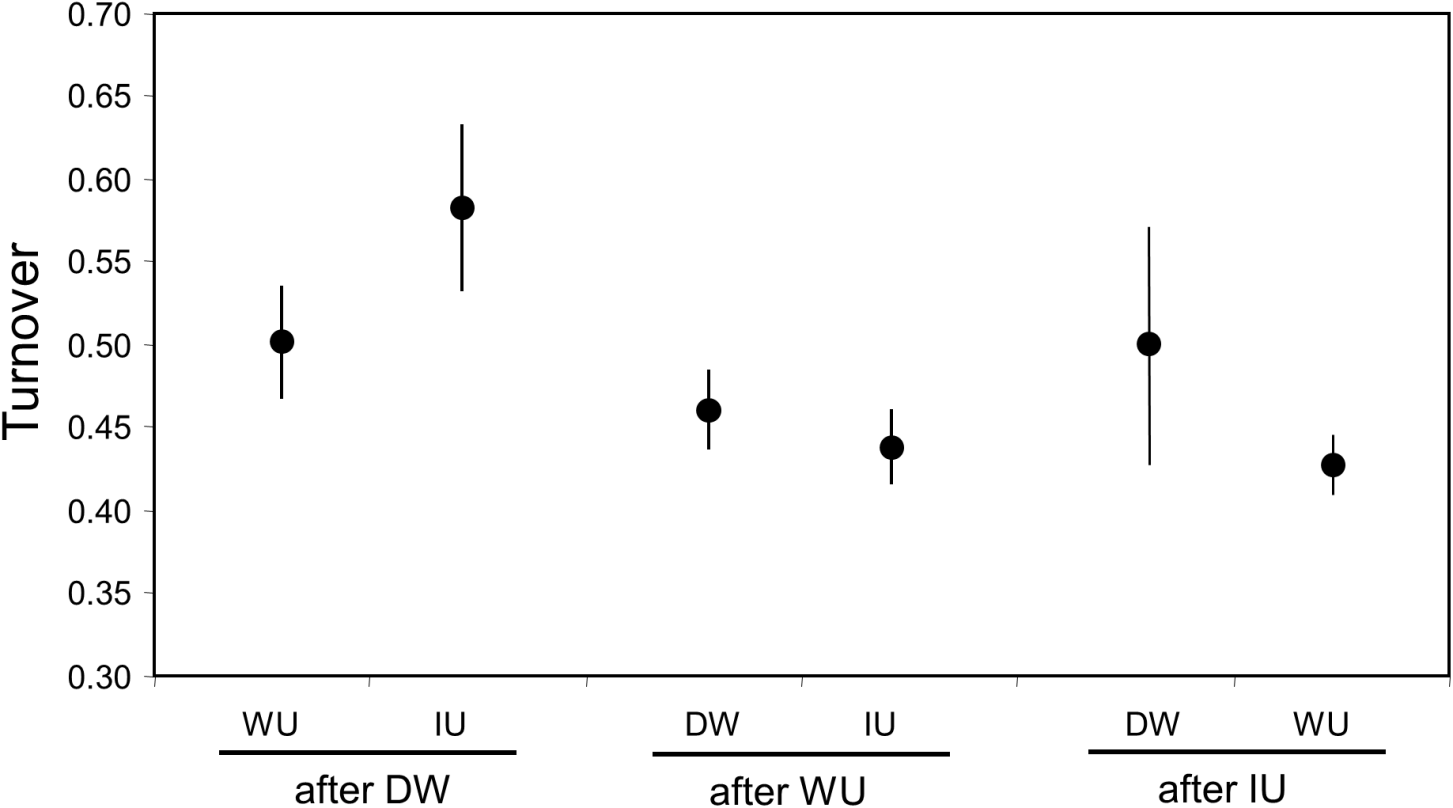
Turnover of the phytoplankton assemblage for changes in the upwelling conditions. Values shown represent the average turnover (20 samples for IU-WU; 18 samples for D-WU; 19 samples for WU-IU; 5 samples for D-IU; 19 samples for WU-D; 4 samples for IU-D) and respective standard error for the six possible alterations in the upwelling. IU: intense upwelling; WU: weak upwelling; D: downwelling.

The short-term turnover values show that the most important changes in the composition of the phytoplankton assemblage took place when a period of IU occurred after a D period (Fig 7). These high turnover values were mainly due to large increases in taxa richness. In the opposite situation, D after IU, turnover values were more variable but generally a relatively high number of taxa was lost from one period to the next. Rapid changes in the oceanographic conditions appear to favour higher turnover of the assemblage as was also illustrated in Fig 4, which showed that the highest seasonal values occurred during the dynamic year of 2005.

Among the taxa that were, on average, dominant in either one of the three groups considered for this analysis there are important changes in ranking: 1) OTUs that decreased their dominance from D to WU to IU, including a variety of haptophytes (*Emiliania huxleyi*, *Gephyrocapsa* spp. A), Bacillariophyceae (*Guinardia delicatula*, centric diatoms A and B, pennate diatoms C, *Pseudo-nitzschia* spp. B) and Dinophyceae (dinoflagellates B and dinoflagelates naked B); 2) OTUs that decreased their dominance from IU to WU to D, mostly Bacillariophyceae (*Leptocylindrus danicus*, *Thalassiosira* spp. C, *Chaetoceros costatum*, *Chaetoceros* spp. A and B, *Pseudo-nitzschia* spp. C, pennate diatoms B) and one haptophyte (*Syracosphaera pulchra*); 3) OTUs that showed their highest dominance during WU periods, including two Dinophyceae (*Prorocentrum minimum*, *Scrippsiella* cf. *trochoidea*) and one Bacillariophyceae (*Pseudo-nitzschia* spp. D). Note that in each of these groups there is a different morphotype of *Pseudo-nitzschia* spp. (see also S1 Table). The taxa that contributed more to the similarity between oceanographic regime groups (S4 Table) were mainly the same frequent OTUs that were already mentioned above for seasonal groups: *L*. *danicus*, *E*. *huxleyi*, *C*. *closterium*; *Paralia sulcata*, centric diatoms A; pennate diatoms A, and *Scrippsiella* cf. *trochoidea*. Each of these OTUs contributed less than 8% to the average similarity within the oceanographic regime groups. The average dissimilarities between the groups varied between 58.6 and 60.3% and again these values resulted from a large number of OTUs with very low individual percentage contributions (<2.2%). *Leptocylindrus danicus*, *Gephyrocapsa* spp. A, centric diatoms B and pennate diatoms C were the most relevant contributors to average dissimilarities between assemblages under different upwelling conditions.

### Linking biological and environmental data

**Fig 8:**
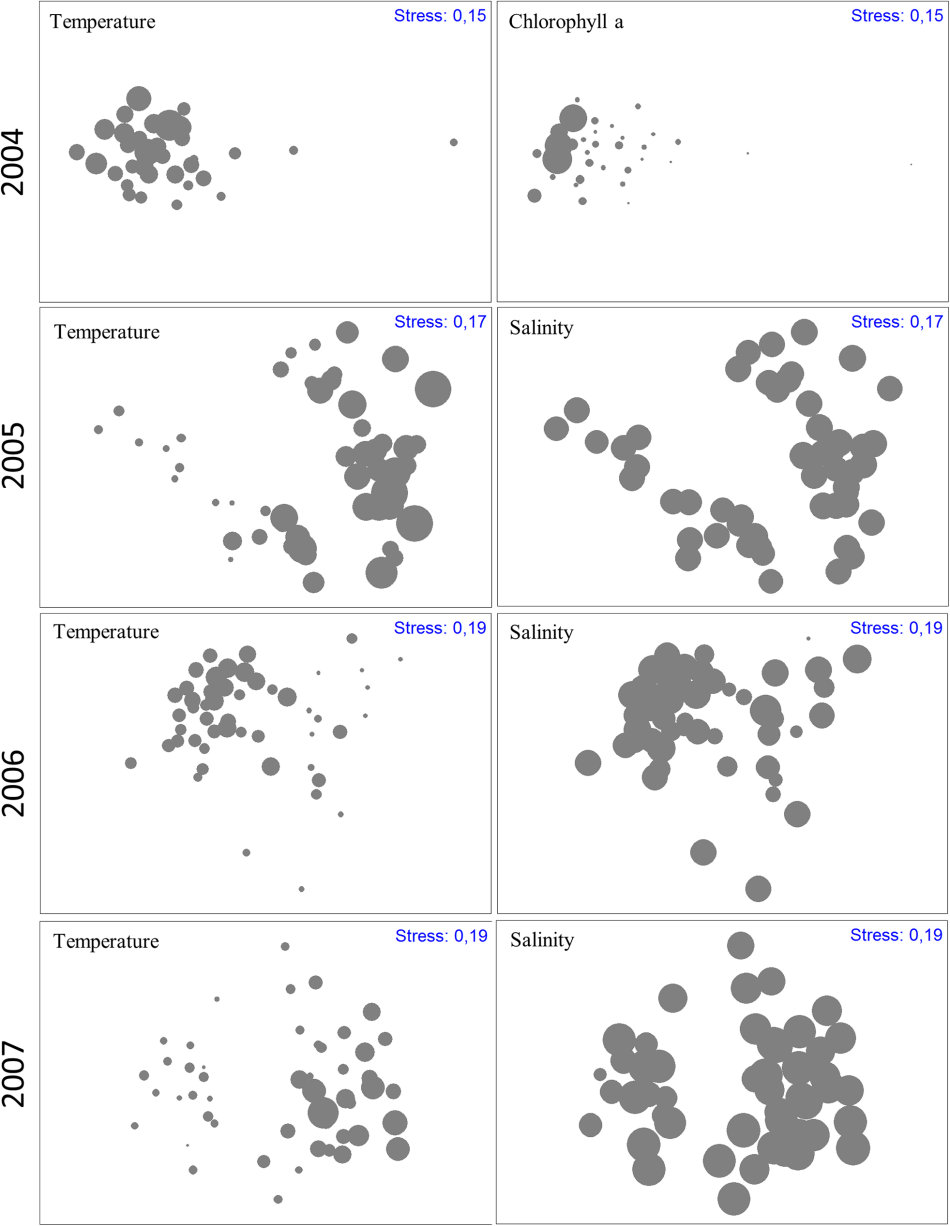
Environmental variables identified by BIO-ENV as the best correlated to the temporal changes in the phytoplankton assemblage. The abiotic variables are superimposed onto the biotic MDS as bubbles with varying sizes; the larger the bubble the greater the value of the superimposed variable.

The changes in the composition and structure of the phytoplankton assemblage throughout the study period were significantly correlated with the fluctuations in the main environmental variables (RELATE: *ρ* = 0.176, p<0.001). The results of the BEST–BIOENV analysis (Table 3, Fig 8) show that proxies for seasonal variation, such as the temperature and salinity, were the most important variables explaining the temporal changes in the phytoplankton assemblage in all studied years. The influence of the oceanographic regime was also important in all years, except for 2006 when less dynamic oceanographic fluctuations were observed. In 2006 the highest Spearman correlation between biological and environmental data was obtained by the combination of the two seasonal proxies only. In the most dynamic years (2005 and 2007), the best correlated upwelling proxy was the maximum upwelling index which supports our previous suggestions that periods of intense upwelling induce important changes in the composition and structure of the phytoplankton assemblage.

**Table 3:** Results of the BIO-ENV analysis showing the environmental variable combinations that yield the highest Spearman correlations with the biological data in each of the four years studied. These combinations are the ones that better explain the observed changes in the phytoplankton assemblages.

Year	Spearman correlation	Significance	Best environmental variable combinations
2004	0.414	0.01**	temperature, chlorophyll a, mean upwelling index
2005	0.391	0.01**	temperature, salinity, chlorophyll a, maximum upwelling index
2006	0.532	0.01**	temperature, salinity
2007	0.316	0.01**	temperature, salinity, tidal range, maximum upwelling index

**: very significant values (p<0.01).

## Discussion

### Seasonal variability of phytoplankton assemblage

The statistical analyses (ANOSIM, RELATE–CYCLICITY) of the results on phytoplankton dynamics at the studied location confirm the overriding importance of seasonality over longer- or shorter-term fluctuations (interannual and upwelling-related, respectively). Seasonality was also pointed as the major source of variability influencing the phytoplankton succession in other studies on the West Iberian Margin (Lisbon Bay: [23]; Galicia: [43–45]).

In temperate regions, the seasonal changes of solar angle and irradiance induce predictable patterns of thermal stratification and light limitation for phytoplankton growth. Basically, the seasonal cycle is driven by sea-surface temperature and the onset of the thermocline leading to phytoplankton blooms during spring, the prevalence of thermal stratification leading to exhaustion of nutrients and subsequent demise of phytoplankton during summer-autumn, and remixing and regeneration of nutrients during winter. In accord, our analyses (BEST–BIOENV) retrieved temperature as the main driver for seasonal change. The seasonal signal was clear in the fluctuations of chlorophyll *a* concentration, ranging from 0.5 to 11 mg.m^-3^ and consistent with values previously recorded in the Iberian Margin (Galician Rías: [46]; Lisbon Bay: [23,47];). Moreover, the seasonal cycle in the phytoplankton assemblage was most evident in the high turnover from the winter assemblage, characterized by the prevalence of the haptophyte *Emiliania huxleyi*, but with low values of taxa richness, abundance, and dominance, to the spring assemblage with high values of these indices and particularly dominated by the diatom *Leptocylindrus danicus*, which accounted for up to 60% of the total abundance.

Spring was mainly characterized by the occurrence of large and/or chain forming diatoms (e.g. *L*. *danicus*, *Chaetoceros* spp.; large *Thalassiosira* spp). Large-sized phytoplankters are typically dominant under nutrient replete conditions necessary to fulfil their growth requisites [48]. During the occurrence of phytoplankton blooms the spring assemblage also included high densities dinoflagellates, especially *Prorocentrum minimum*., which probably takes advantages of a versatile nutrition strategy involving mixotrophy [49].

When relatively stable conditions persisted, as during the spring-summer of 2006, the peak of chlorophyll in the <20 μm fraction of the plankton indicated a biomass increase in nano- or picoplankton, with the contribution of unlisted groups, such as cryptophytes and small green algae. A marked increase of small-sized phytoplankters (with high contributions of *Emiliania huxleyi* and other haptophytes, Table 3), also reported by other authors (e.g. [12,50]), is consistent with the advantage that a large surface-to-volume ratio provides in the low nutrient and high irradiance conditions that are common in the upper level of a stratified water column [51,52], especially during summer and autumn. The persistence of stratified conditions is also known to favour dinoflagellates (e.g. the genera *Ceratium*, *Dinophysis*, *Protoperidinium*, *Gymnodinium*, *Gyrodinium* and *Prorocentrum*) which compete in grazing with ciliates and other heterotrophic species, eventually reducing chlorophyll a concentrations.

During winter, total chlorophyll a concentration was low as decreasing temperature and solar irradiance, together with high turbulence and water mixing, made conditions unfavourable for the phytoplankton to thrive. Small cells have a superior capacity to acquire nutrients even at low light conditions [48] and therefore the winter phytoplankton assemblage was characterized by the prevalence of the haptophyte *Emiliania huxleyi* and low abundances of perennial small centric diatoms. Occasionally also pennate diatom species occurred, likely resuspended by turbulence from the benthos (especially *Cylindrotheca closterium* and ‘diatoms, pennate A’).

### Upwelling influence on phytoplankton assemblage

Seasonality has been proved to play a role in providing the general conditions, especially of light and temperature, that will promote or limit the development of phytoplankton assemblages under upwelling [53,54]. In temperate regions, upwelling events disrupt the seasonal cycle of thermal stratification inducing changes in the phytoplankton assemblage. Phytoplankters adapt to different combinations of nutrient concentration, light availability and turbulence intensity [55,56], and general trends at the higher taxonomic level (e.g. diatoms vs. dinoflagellates) are often observed (e.g. [3,57–59]). Diatoms are in general well adapted to turbulent, nutrient-rich environments resulting from the advection of deep waters while flagellated species are stronger competitors under nutrient-poor, stratified conditions [60,61]. Yet, the complex interaction of environmental factors makes short-term variability in phytoplankton composition nearly unpredictable (e.g. [28]). Our results indicate that both the frequency of events and intensity of physical forcing are important drivers of such variability, but the outcome in terms of species composition is highly dependent on the available local pool of species and the timing in relation to the seasonal cycle. Silva et al. [23] also observed year-to-year changes in the Lisbon Bay phytoplankton assemblage according to the duration and strength of the upwelling events, while Du and Paterson [58] report differences in phytoplankton community structure (California) related to when a sample was collected within an upwelling/downwelling cycle rather than to the strength of upwelling within a given year. Our results showed changes in species composition and community structure that were less dramatic when oceanographic conditions were less variable (year 2006) or under light-limiting conditions during the winter (all years, Fig 6). The estimated short-term species turnover related to changes in upwelling-downwelling conditions was generally higher than the seasonal turnover. Therefore, different species from the seasonally available pool may thrive under upwelling or downwelling conditions, but these species were not the same during every season or year. Besides a few prevalent species that may be considered as indicators of upwelling or downwelling, the composition and structure of the assemblages under similar oceanographic conditions was highly variable from season-to-season and from year-to-year and was likely determined by the occasional local availability of species with the adequate biological traits (e.g. growth rate, functional type, physical and chemical requisites to thrive under these particular oceanographic conditions.

Fast-growing small species of *Chaetoceros*, *Thalassiosira* and *Skeletonema*, are typical of the very early stage of upwelling, followed by larger diatoms such as *Lauderia* and *Thalassiosira nitzschioides* [23,47,62]. However, at species level, the dominant diatoms found in our study and previous studies (e.g. [58,63]) vary with season or from year to year. In our samples, the chain-forming *Leptocylindrus* was much more frequently abundant than chains of *Skeletonema*, perhaps as a result of direct competition. The predominance of small-sized diatom species, in IU agrees with the dependence that these non-motile, relatively abundant organisms have on turbulence for resuspension in the euphotic zone where their can efficiently utilize the abundance of nutrients typical of upwelling waters for rapid cell number increase [54].

It is noteworthy that *Prorocentrum minimum* and *Scrippsiella* cf. *trochoidea*, both giving sizeable contributions to WU assemblages, were grouped together in “bloom and vegetation life-form” type II, for which the eutrophic Oslofjord was given as a typical site [56]. These species, along with other undetermined, generally small, dinoflagellates seem to benefit from the low to moderate advection conditions during WU, and perhaps also from the abundance of small plankton cells that may serve as food for mixotrophic dinoflagellates [49,64]. *Scrippsiella* cf. *trochoidea* and several other dinoflagellates were also present under IU conditions reported here, indicating their capability of growing under conditions of higher turbulence than originally envisioned by Margalef [3], as pointed out by Smayda [55].

The haptophytes *Emiliania huxleyi* and *Gephyrocapsa* spp. were major contributors to communities associated with the relaxation periods (D). *Dinophysis*, *Protoperidinium*, *Ceratium* and other dinoflagellates also occurring under D conditions, are generally associated with the third stage of succession in a model proposed by Palma et al. [47] for Tagus estuary, and probably favoured by traits like motility and mixotrophy facilitating the acquisition of nutrients for growth under the nutrient-poor, stratified conditions during relaxation periods (e.g. [60,61]).

### Potentially harmful algae

Associated with the elevated productivity, phytoplankton blooms often included noxious phytoplankton resulting in the toxification of shellfish. Recurrent episodes of toxin accumulation in bivalve farms of Ria de Aveiro have been associated with the occurrence of *Dinophysis* species, especially *D*. *acuminata* and *D*. *acuta* [20]. Both species are highly motile, mixotrophic organisms, able to take advantage of repeated changes in oceanographic regime [55]. Their occurrence in concentrations high enough to cause toxic outbreaks has been associated with two sets of environmental conditions: periods of stratification between moderate pulses of upwelling, during which they multiply in a diatom-rich water column; or accumulation during downwelling events [65]. In our samples *Dinophysis* occurred with the highest abundance during the summer of 2005 (S3 Table), mostly associated to periods of low to moderate advection (WU, S4 Table). An episode of paralytic shellfish toxin accumulation in Ria de Aveiro bivalve farms during the autumn 2007 also involved the chain forming dinoflagelate *Gymnodinium catenatum* and was compatible with the occurrence of this species in our samples. In previous outbreaks, *G*. *catenatum* was shown to be transported northward along the coast from the Lisbon area, eventually entering Ria de Aveiro and extending further north into the Galician coast [66] and similar events have been reported for blooms of *Dinophysis* [67].

‘*Pseudo-nitzschia* spp. A’ appeared strongly associated with the spring assemblage, apparently taking advantage of increased turbulence and higher nutrient levels, and were usually a very relevant group after the first diatom bloom in early spring. These results are consistent with the global model and with the models by season of Palma et al. [47], which placed *Pseudo-nitzschia* spp. in the second step of the succession after 4–6 days of intensification of an upwelling event. Similar observations were reported from the Galician Rías [62]. As it was suggested for the Tagus river nutrient input in Lisbon Bay [47], the supply of nutrients from Ria de Aveiro is likely to play an important role in stimulating toxic *Pseudo-nitzschia* blooms, causing Amnesic Shelfish Poisoning (ASP) outbreaks on local shellfish farms relatively early in the year. Sustained high levels of nutrient introduction have the potential to change the balance between N and P [68], thereby affecting the composition of the phytoplankton community, and sometimes promoting the growth of harmful algae [69,70]. The typically multispecific blooms of *Pseudo-nitzschia* that occur on the west Iberian margin are usually dominated by the *seriata*-group (larger forms) in the spring and by the *delicatissima*-group (smaller forms) during summer [47,71]. However, an opposite trend has also been reported by Fehling et al. [72] in Western Scottish coastal waters and *Pseudo-nitzschia australis*, of the *seriata*-group, was found the most abundant of a diverse assemblage of *Pseudo-nitzschia* species in Ria de Aveiro during August 2000 [73].

## Conclusions

Long-term series on the composition of species-rich phytoplankton assemblages are difficult to produce, owing to taxonomical and methodological constraints (e.g. taxa that cannot be identified to species level by light microscopy of preserved samples; [74], changes in operators that may compromise data comparability). The samples studied herein were consistently handled using standardized procedures and provided a four-year time-series of weekly quantitative data, albeit with various levels of taxonomic resolution. This most probably lead to underestimated species numbers but using all of the available data is still the best option, since discarding or aggregating parts of the data means losing biodiversity information [75].

In this study we have identified a total of 315 taxa included in five major lineages a number comparable to the 300 phytoplankton taxa reported from the California Current eastern boundary upwelling system [76]. This large dataset allowed estimation of several diversity indices (rarely applied to phytoplankton studies) and address the links between phytoplankton assemblage dynamics and major environmental factors. In fact, most studies on phytoplankton dynamics focus on the fluctuations of higher taxonomic groups, such as diatoms vs dinoflagelates, a separation that is not sufficient to explain small-scale changes, like the ones observed under dynamic upwelling regimes.

Despite the plethora of studies on upwelling its relationship to changes in phytoplankton composition is seldom tested statistically. Defining threshold values to differentiate three categories of oceanographic conditions was fundamental in our analyses for demonstrating statistically significant differences in phytoplankton assemblages associated to D, WU and IU. Bakun values of –300 to –400 m^3^km^-1^s^-1^, close to our proposed threshold for IU events (–500 m^3^km^-1^s^-1^), were also indicated as the intensity of spring upwelling events in Galicia, necessary to induce the typical changes in the dominant phytoplankton species [53].

Repeated cycles of upwelling and relaxation result in an overall higher productivity (and subsequently also carbon export and sequestration in the sediments) than long periods of stratification that usually lead to nutrient exhaustion [77,78]. Although it is clear that phytoplankton diversity and productivity are not coupled on all time-scales [48], different taxa contribute differently to the production and respiration and export of organic carbon [79]. Our study shows that the duration, frequency and intensity of upwelling events, which vary seasonally and inter-annually, is paramount for maintaining long-term phytoplankton diversity by allowing unstable coexistence and by incorporating species turnover at different scales.

Although the time series of four years weekly sampling provides a solid basis for the detection of seasonal and inter-annual variation in phytoplankton assemblages, the observed variation in physical forcing was notable among the studied years (2004 to 2007) and longer series are needed before long-term trends in biodiversity can be reliably recognized. However, several authors recognised a contemporary trend for a decrease in intense upwelling events and increased downwelling periods (Portuguese coast [80], Galicia [81] and California [82]). Such a global decrease in frequency and intensity of coastal upwelling events may have important socio-economic consequences [11] both by the decline of fisheries and by increasing the frequency of HAB.

By providing detail on the species composition and several biodiversity and community structure indices our results contribute to the understanding of the complex mechanisms of coastal phytoplankton dynamics and productivity in relation to changing physical forcing. This knowledge is fundamental to improve predictability of future prospects under climate changes.

## Supporting information

S1 Fig MDS results of the analysis performed on the phytoplankton data from all samples collected; stress value: 0.24. (A) Samples coded by year; (B) Samples coded by season.
(TIF)

S2 Fig MDS results of the analysis performed on the phytoplankton data from all samples collected; stress value: 0.24. Samples are coded according to the observed values of the upwelling index.
(TIF)

S1 Table List of operational taxonomic units (OTUs) used for organisms unidentifiable at species level, and then grouped within a genus (only genera with size classes defined are included), or at genus level (and then grouped under class- or phyllum-level groups). Size classes were distinguished in most cases. Groups are listed in the following order: Bacillariophyceae (diatoms), Dinophyta (dinoflagellates), Euglenophyta, Haptophyta and Prasinophyceae.
(DOC)

S2 Table %CUM represent the cumulative percentage of most abundant species per season in studied years. Species with less than 0.01% of the total abundance were discarded.
(XLS)

S3 Table Breakdown of percentual contributions from SIMPER analysis for comparisons between assemblages sampled in different seasons (all years combined). The taxa listed contribute at least 1.2%.
(DOC)

S4 Table Breakdown of percentual contributions from SIMPER analysis for comparisons between assemblages sampled during different oceanographic conditions (all years combined). The taxa listed contribute at least 1%.
(DOC)
