# Supplementary figures and images for: Phytoplankton dynamics in relation to seasonal variability and upwelling and relaxation patterns at the mouth of Ria de Aveiro (West Iberian Margin) over a four-year period
Source: PLoS One. 2017 May 4;12(5):e0177237. doi: 10.1371/journal.pone.0177237 (PMC5417713; doi:10.1371/journal.pone.0177237)

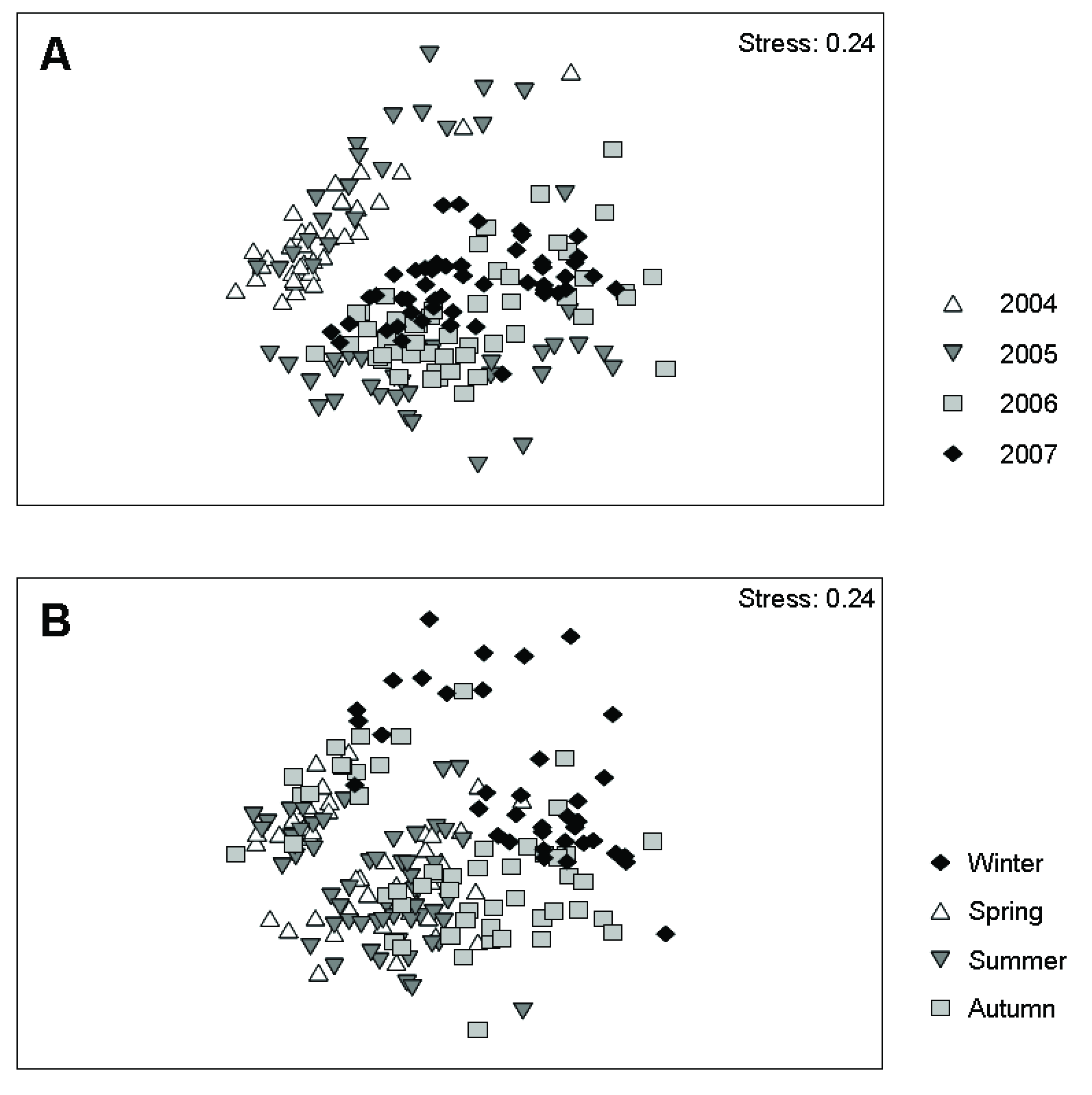

Supplement: S1 Fig — (A) Samples coded by year; (B) Samples coded by season. (TIF) [file pone.0177237.s001.tif]

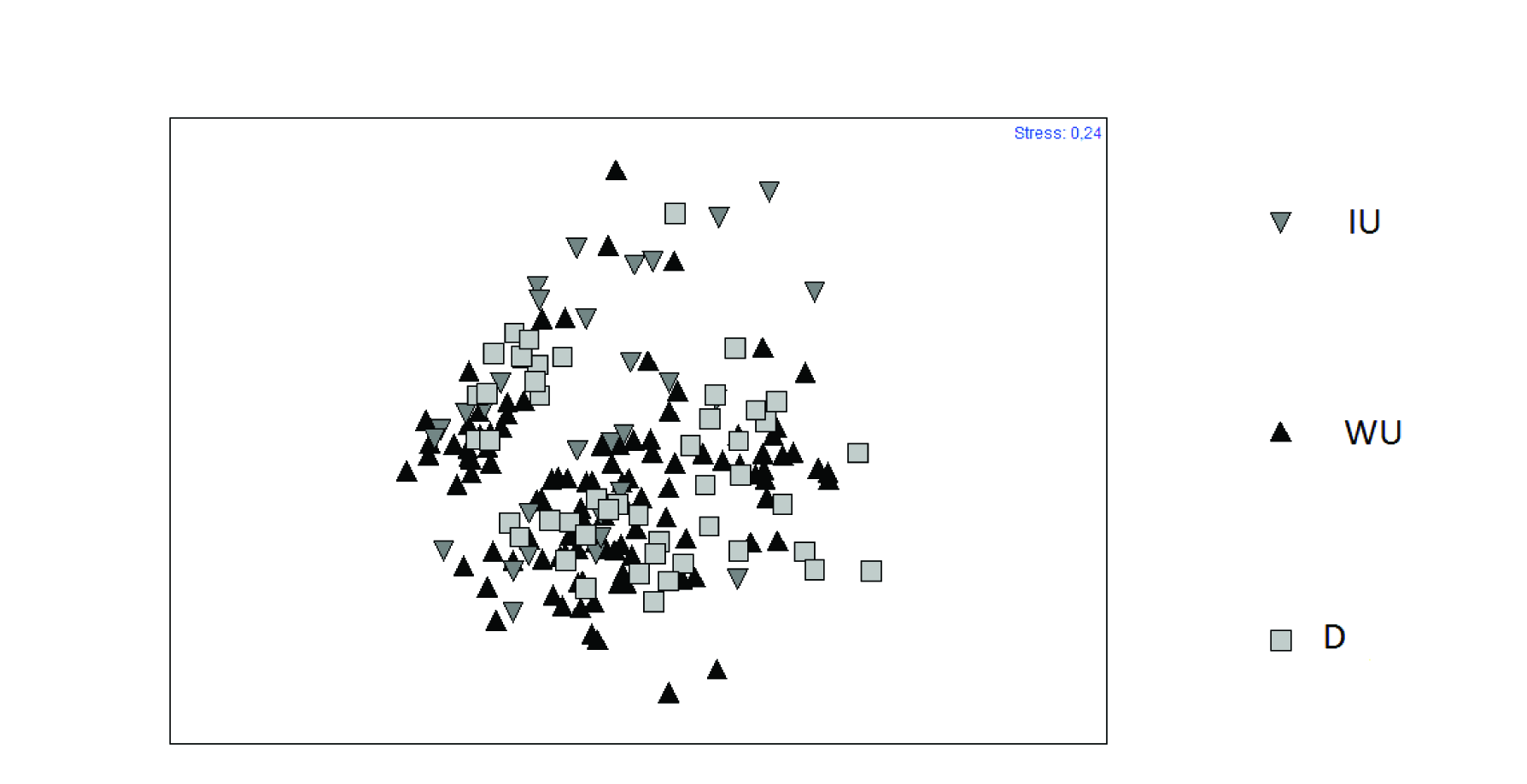

Supplement: S2 Fig — Samples are coded according to the observed values of the upwelling index. (TIF) [file pone.0177237.s002.tif]
