# Supplementary material for: Phytoplankton dynamics in relation to seasonal variability and upwelling and relaxation patterns at the mouth of Ria de Aveiro (West Iberian Margin) over a four-year period
Source: PLoS One. 2017 May 4;12(5):e0177237. doi: 10.1371/journal.pone.0177237 (PMC5417713; doi:10.1371/journal.pone.0177237)
Supplement: S1 Table — Size classes were distinguished in most cases. Groups are listed in the following order: Bacillariophyceae (diatoms), Dinophyta (dinoflagellates), Euglenophyta, Haptophyta and Prasinophyceae. (DOC) [file pone.0177237.s003.doc]

S1 Table. List of operational taxonomic units (OTUs) used for organisms unidentifiable at species level, and then grouped within a genus (only genera with size classes defined are included), or at genus level (and then grouped under class- or phyllum-level groups). Size classes were distinguished in most cases. Groups are listed in the following order: Bacillariophyceae (diatoms), Dinophyta (dinoflagellates), Euglenophyta, Haptophyta and Prasinophyceae.

| OTU | counted items |
| --- | --- |
| *Chaetoceros* spp. A | valve diameter > 15 µm |
| *Chaetoceros* spp. B | valve diameter 5–15 µm |
| *Chaetoceros* spp. C | valve diameter < 5 µm |
| *Pseudo-nitzschia* spp. A | valve width > 5 µm |
| *Pseudo-nitzschia* spp. B | valve width ≈ 5 µm |
| *Pseudo-nitzschia* spp. C | valve width < 5 µm |
| *Pseudo-nitzschia* spp. D | all sizes (used for first 9 months of counting period) |
| *Thalassiosira* spp. A | valve diameter > 20 µm |
| *Thalassiosira* spp. B | valve diameter 10–20 µm |
| *Thalassiosira* spp. C | valve diameter 5–10 µm |
| *Thalassiosira* spp. D | valve diameter < 5 µm |
| diatoms, centric A | undetermined centrics with valve diameter > 5 µm |
| diatoms, centric B | undetermined centrics with valve diameter < 5 µm |
| diatoms, centric C | undetermined centrics attached to suspended particles |
| diatoms, pennate A | undetermined pennates with longest axis > 30 µm |
| diatoms, pennate B | undetermined pennates with longest axis 5–30 µm |
| diatoms, pennate C | undetermined pennates with longest axis < 5 µm |
| dinoflagellates A | undetermined dinoflagellates, theca uncertain, > 20 µm |
| dinoflagellates B | undetermined dinoflagellates, theca uncertain, < 20 µm |
| dinoflagellates naked A | undetermined naked dinoflagellates > 20 µm |
| dinoflagellates naked B | undetermined naked dinoflagellates < 20 µm |
| dinoflagellates thecate A | undetermined thecate dinoflagellates < 20 µm |
| Euglenophyceae und. | undetermined euglenoids |
| *Gephyrocapsa* spp. A | cell diameter > 5 µm |
| *Gephyrocapsa* spp. B | cell diameter < 5 µm |
| *Pyramimonas* spp. A | cell diameter ≈ 5 µm |
| *Pyramimonas* spp. B | cell diameter < 5 µm |
