# Supplementary material for: Phytoplankton dynamics in relation to seasonal variability and upwelling and relaxation patterns at the mouth of Ria de Aveiro (West Iberian Margin) over a four-year period
Source: PLoS One. 2017 May 4;12(5):e0177237. doi: 10.1371/journal.pone.0177237 (PMC5417713; doi:10.1371/journal.pone.0177237)
Supplement: S3 Table — The taxa listed contribute at least 1.2%. (DOC) [file pone.0177237.s005.doc]

S3 Table. Breakdown of percentual contributions from SIMPER analysis for comparisons between assemblages sampled in different seasons (all years combined). The taxa listed contribute at least 1.2% during some of the seasons. Numbers in bold mark the six dominant species in each season.

W: winter; Sp: spring; Su: summer; A: autumn; AS: average similarity; AD: average dissimilarity; ●: contributions lower than 1.2%.

|  | Density (cells.100ml^-1^) | | | |  | % Contribution | | | |  | % Contribution | | | | | |
| --- | --- | --- | --- | --- | --- | --- | --- | --- | --- | --- | --- | --- | --- | --- | --- | --- |
|  | Sp | Su | A | W |  | Sp | Su | A | W |  | Sp/Su | Sp/A | Sp/W | Su/A | Su/W | A/W |
| Total | 35666.9 | 35640.7 | 14147.4 | 5514.3 | AS: | 46.8 | 48.1 | 44.0 | 42.3 | AD: | 54.3 | 58.7 | 64.2 | 57.8 | 65.8 | 61.7 |
| **Bacillariophyceae** |  |  |  |  |  |  |  |  |  |  |  |  |  |  |  |  |
| diatoms, centric A | 113.1 | 138.2 | 266.2 | 105.9 |  | 4.48 | 3.73 | 5.64 | 7.97 |  | ● | ● | ● | ● | ● | ● |
| diatoms, centric B | 315.6 | 254.2 | **764.8** | 97.4 |  | 1.48 | ● | 3.87 | 1.44 |  | 1.63 | 1.77 | 1.67 | 1.78 | 1.54 | 2.24 |
| diatoms, centric C | 12.3 | 2.9 | 170.0 | 2.0 |  | ● | ● | ● | ● |  | ● | ● | ● | ● | ● | 1.22 |
| diatoms, pennate A | 104.6 | 156.2 | 255.1 | 149.6 |  | 4.72 | 4.12 | 6.11 | 9.09 |  | ● | ● | ● | ● | ● | ● |
| diatoms, pennate B | 97.8 | 1187.5 | 67.0 | 33.4 |  | ● | ● | ● | ● |  | 1.32 | ● | ● | ● | ● | ● |
| diatoms, pennate C | 220.0 | 226.0 | 507.8 | 166.7 |  | 1.45 | ● | 3.47 | 2.80 |  | 1.58 | 1.71 | 1.71 | 1.72 | 1.61 | 2.04 |
| *Cerataulina pelagica* | 679.0 | 57.4 | 10.8 | 12.4 |  | ● | ● | ● | ● |  | 1.24 | ● | 1.25 | ● | ● | ● |
| *Chaetoceros* spp. A | **1270.4** | 319.1 | 76.6 | 192.0 |  | 2.92 | 2.47 | 1.38 | 1.58 |  | 1.51 | 1.60 | 1.87 | 1.51 | 1.69 | 1.62 |
| *Chaetoceros* spp. B | 164.9 | **1417.4** | 104.2 | **233.9** |  | ● | ● | ● | ● |  | ● | ● | ● | ● | ● | ● |
| *Chaetoceros* spp. C | 184.4 | 104.5 | 521.7 | **266.7** |  | ● | ● | ● | ● |  | ● | 1.30 | ● | 1.37 | ● | 1.65 |
| *Cylindrotheca closterium* | 142.6 | 325.5 | 251.5 | **198.5** |  | 3.52 | 4.10 | 4.96 | 7.04 |  | ● | ● | ● | ● | ● | 1.21 |
| *Detonula pumila* | 517.9 | 743.7 | 15.7 | 137.1 |  | 1.38 | ● | ● | ● |  | 1.55 | 1.46 | 1.67 | ● | 1.24 | 1.28 |
| *Guinardia* cf. *delicatula* | 300.3 | 45.3 | 135.7 | 185.9 |  | 1.94 | ● | 2.82 | 1.56 |  | 1.41 | 1.30 | 1.57 | 1.38 | 1.22 | 1.71 |
| *Guinardia* cf. *striata* | **1328.8** | 117.1 | 23.1 | 12.4 |  | ● | ● | ● | ● |  | 1.44 | 1.31 | 1.40 | ● | ● | ● |
| *Leptocylindrus danicus* | **14237.3** | **8341.3** | **1388.1** | 59.6 |  | 5.95 | 6.16 | 4.86 | ● |  | 1.67 | 1.89 | 3.13 | 1.63 | 2.92 | 2.38 |
| *Meuniera*? | 32.2 | 4.3 | 27.2 | 24.7 |  | ● | ● | ● | ● |  | ● | ● | ● | ● | ● | 1.25 |
| *Odontella mobiliensis* | 5.5 | 2.2 | 5.3 | 12.5 |  | ● | ● | ● | 1.55 |  | ● | ● | ● | ● | ● | ● |
| *Paralia sulcata* | 100.6 | 86.6 | 142.7 | 186.4 |  | 2.15 | 1.85 | 4.65 | 9.02 |  | 1.28 | 1.22 | 1.34 | 1.22 | 1.29 | ● |
| *Pleurosigma* spp. | 7.5 | 6.3 | 3.3 | 8.1 |  | ● | ● | ● | 1.53 |  | ● | ● | ● | ● | ● | ● |
| *Proboscia alata* | 37.0 | 339.9 | 9.0 | 0.6 |  | ● | 3.10 | ● | ● |  | 1.63 | ● | ● | 1.72 | 2.01 | ● |
| *Pseudo-nitzschia* spp. A | **1148.8** | 410.2 | 331.6 | 48.7 |  | 1.74 | ● | ● | ● |  | 1.69 | 1.69 | 1.77 | 1.48 | 1.27 | 1.54 |
| *Pseudo-nitzschia* spp. C | 293.7 | **3141.5** | 122.8 | 63.7 |  | ● | 2.10 | ● | 2.27 |  | 1.85 | 1.43 | 1.55 | 1.77 | 1.79 | 1.58 |
| *Pseudo-nitzschia* spp. D | 770.4 | 1260.3 | 6.0 | 15.7 |  | ● | ● | ● | ● |  | 1.38 | ● | ● | ● | ● | ● |
| *Skeletonema* sp. | 15.6 | 0.4 | 27.6 | **377.4** |  | ● | ● | ● | ● |  | ● | ● | ● | ● | ● | 1.29 |
| *Thalassionema nitzschioides* | 24.6 | 804.9 | 103.3 | 0.5 |  | ● | ● | ● | ● |  | ● | ● | ● | 1.22 | ● | ● |
| *Thalassiosira* spp. B | 96.4 | 87.2 | 16.2 | 138.4 |  | ● | ● | ● | ● |  | ● | ● | 1.24 | ● | ● | ● |
| *Thalassiosira* spp. C | 165.3 | **1679.5** | 103.2 | 65.0 |  | ● | ● | ● | 1.47 |  | 1.36 | ● | 1.33 | 1.27 | 1.36 | 1.38 |
| **Chlorophyta** |  |  |  |  |  |  |  |  |  |  |  |  |  |  |  |  |
| *Pediastrum sp.* | 5.7 | 1.0 | 8.9 | 43.3 |  | ● | ● | ● | 1.49 |  | ● | ● | ● | ● | ● | 1.22 |
| **Dinophyceae** |  |  |  |  |  |  |  |  |  |  |  |  |  |  |  |  |
| dinoflagellates A | 92.3 | 94.2 | 90.9 | 18.1 |  | 3.60 | 3.07 | 3.38 | 3.85 |  | ● | ● | ● | ● | ● | ● |
| dinoflagellates B | **1492.9** | 1017.4 | **591.8** | 53.9 |  | ● | ● | ● | ● |  | 1.78 | 1.74 | 1.70 | 1.41 | ● | 1.24 |
| dinoflagellates naked A | 163.7 | 542.1 | 71.9 | 31.4 |  | ● | ● | ● | ● |  | 1.49 | 1.29 | 1.34 | 1.35 | 1.33 | 1.20 |
| dinoflagellates naked B | 351.3 | 493.9 | 371.1 | 25.4 |  | 2.57 | 2.39 | ● | 2.21 |  | 1.66 | 1.73 | 1.78 | 1.75 | 1.75 | 1.52 |
| dinoflagellates thecate A | 80.7 | 180.0 | 33.4 | 5.3 |  | ● | ● | ● | ● |  | 1.36 | ● | 1.21 | 1.25 | 1.24 | ● |
| *Ceratium furca* | 20.2 | 75.8 | 59.3 | 0.4 |  | ● | 1.47 | 1.23 | ● |  | ● | ● | ● | ● | 1.20 | ● |
| *Ceratium fusus* (300 µm) | 63.1 | 200.4 | 108.4 | 2.0 |  | 1.82 | 3.25 | 1.38 | ● |  | 1.25 | ● | 1.39 | 1.40 | 1.96 | 1.28 |
| *Dinophysis acuta* | 3.7 | 253.8 | 93.9 | 0.0 |  | ● | ● | ● | --- |  | ● | ● | ● | 1.26 | ● | ● |
| *Diplopsalis* sp. | 64.4 | 45.4 | 116.4 | 2.6 |  | 3.46 | 2.26 | ● | ● |  | ● | 1.27 | 1.59 | ● | 1.21 | ● |
| *Gymnodinium* spp. | 22.5 | 111.7 | 26.0 | 4.5 |  | 1.48 | 1.27 | ● | ● |  | ● | ● | ● | ● | ● | ● |
| *Gymnodinium catenatum* | 5.7 | 15.4 | 156.5 | 0.4 |  | ● | ● | ● | ● |  | ● | ● | ● | ● | ● | 1.23 |
| *Gyrodinium* spp. | 71.9 | 55.8 | 9.3 | 4.1 |  | 1.81 | ● | ● | ● |  | ● | ● | 1.24 | ● | ● | ● |
| *Gyrodinium fusiforme* | 82.1 | 180.4 | 83.4 | 24.9 |  | 3.94 | 3.33 | 2.80 | 2.41 |  | ● | ● | 1.31 | ● | 1.29 | 1.33 |
| *Gyrodinium lacryma* | 43.8 | 24.9 | 5.9 | 4.7 |  | 1.94 | 1.26 | ● | ● |  | ● | ● | 1.31 | ● | ● | ● |
| *Prorocentrum micans* | 16.4 | 50.1 | 19.8 | 0.6 |  | ● | 1.88 | ● | ● |  | ● | ● | ● | ● | 1.38 | ● |
| *Prorocentrum minimum* | **4850.7** | 136.4 | 21.5 | 12.5 |  | 3.10 | 2.23 | ● | ● |  | 1.55 | 1.78 | 2.05 | 1.22 | 1.37 | ● |
| *Protoperidinium bipes* | 31.0 | 20.1 | 4.3 | 3.1 |  | 1.45 | ● | ● | ● |  | ● | ● | ● | ● | ● | ● |
| *Protoperidinium diabolum* | 15.7 | 23.0 | 6.9 | 0.1 |  | ● | 1.97 | ● | ● |  | ● | ● | ● | ● | 1.30 | ● |
| *Protoperidinium divergens* | 9.0 | 26.7 | 9.8 | 0.2 |  | ● | 2.38 | ● | ● |  | ● | ● | ● | ● | 1.43 | ● |
| *Protoperidinium steinii* | 15.3 | 17.6 | 18.8 | 0.7 |  | ● | 1.62 | ● | ● |  | ● | ● | ● | ● | ● | ● |
| *Scripsiella* cf. *trochoidea* | 571.2 | 821.8 | 216.6 | 99.1 |  | 5.82 | 4.67 | 2.88 | 5.19 |  | ● | 1.50 | 1.51 | 1.32 | 1.25 | 1.36 |
| **Euglenophyceae** |  |  |  |  |  |  |  |  |  |  |  |  |  |  |  |  |
| Euglenophyceae und. | 51.7 | 21.5 | 18.0 | 38.1 |  | 1.97 | ● | 1.83 | 2.27 |  | ● | ● | ● | ● | ● | ● |
| **Haptophyta** |  |  |  |  |  |  |  |  |  |  |  |  |  |  |  |  |
| *Emiliania huxleyi* | 1062.2 | **4078.0** | **2468.6** | **1107.7** |  | 3.54 | 5.59 | 8.74 | 11.38 |  | 1.81 | 1.67 | 1.64 | 1.26 | 141 | 1.34 |
| *Gephyrocapsa* spp. A | 815.7 | 1362.0 | **1302.2** | **235.9** |  | 1.90 | 2.95 | 4.69 | 2.63 |  | 1.94 | 1.98 | 1.95 | 1.8 | 1.97 | 2.45 |
| *Gephyrocapsa* spp. B | 231.1 | 148.0 | 349.0 | 59.0 |  | ● | ● | ● | ● |  | ● | 1.24 | ● | ● | ● | 1.38 |
| *Syracosphaera pulchra* | 2.0 | **1903.4** | **1738.5** | 0.4 |  | ● | ● | ● | ● |  | ● | ● | ● | ● | ● | ● |
|  |  |  |  |  |  |  |  |  |  |  |  |  |  |  |  |  |
| % Contribution of selected taxa | 91.5 | 93.0 | 95.1 | 82.9 |  | 70.1 | 69.2 | 64.7 | 78.8 |  | 35.4 | 30.9 | 41.5 | 32.1 | 38.0 | 39.0 |
