# Supplementary material for: Phytoplankton dynamics in relation to seasonal variability and upwelling and relaxation patterns at the mouth of Ria de Aveiro (West Iberian Margin) over a four-year period
Source: PLoS One. 2017 May 4;12(5):e0177237. doi: 10.1371/journal.pone.0177237 (PMC5417713; doi:10.1371/journal.pone.0177237)
Supplement: S4 Table — The taxa listed contribute at least 1%. (DOC) [file pone.0177237.s006.doc]

S5 Table. Breakdown of percentual contributions from SIMPER analysis for comparisons between assemblages sampled during different oceanographic conditions (all years combined). The taxa listed contribute at least 1%. Numbers in bold mark the six dominant species in each group.

D: Downwelling; WU: Weak Upwelling; IU: Intense Upwelling; AS: average similarity; AD: Average dissimilarity; ●: contributions lower than 1%.

|  | Density (cells.100ml^-1^) | | |  | % Contribution | | |  | % Contribution | | |
| --- | --- | --- | --- | --- | --- | --- | --- | --- | --- | --- | --- |
|  | D | WU | IU |  | D | WU | IU |  | D/WU | D/IU | WU/IU |
| Total | 9466.1 | 25035.3 | 38996.3 | AS | 43.1 | 41.8 | 40.7 | AD | 58.6 | 59.4 | 60.3 |
| **Bacillariophyceae** |  |  |  |  |  |  |  |  |  |  |  |
| diatoms, centric A | 185.2 | 138.3 | 200.1 |  | 6.46 | 5.18 | 5.51 |  | ● | ● | ● |
| diatoms, centric B | **706.4** | 254.4 | 300.0 |  | 4.35 | 1.47 | 1.34 |  | 1.97 | 1.88 | 1.61 |
| diatoms, pennate A | 168.9 | 164.7 | 187.4 |  | 6.81 | 5.64 | 6.47 |  | ● | ● | ● |
| diatoms, pennate B | 85.5 | 79.5 | **1805.6** |  | ● | ● | ● |  | 1.16 | 1.29 | 1.22 |
| diatoms, pennate C | **557.5** | 198.1 | 200.0 |  | 3.82 | 1.98 | 1.3 |  | 1.90 | 1.84 | 1.57 |
| *Cerataulina pelagica* | 19.9 | 253.6 | 205.8 |  | ● | ● | ● |  |  | 1.13 | 1.13 |
| *Chaetoceros* spp. A | 153.5 | 253.4 | 1619.1 |  | 1.76 | 2.28 | 2.69 |  | 1.64 | 1.61 | 1.57 |
| *Chaetoceros* spp. B | 127.1 | 62.6 | **2582.5** |  | ● | ● | ● |  | ● | 1.01 | ● |
| *Chaetoceros* spp. C | 138.3 | 185.4 | 780.4 |  | ● | ● | ● |  | 1.14 | 1.40 | 1.39 |
| *Coscinodiscus* spp. | 3.3 | 3.4 | 9.4 |  | ● | ● | 1.00 |  | ● | ● | ● |
| *Cylindrotheca closterium* | 141.5 | 215.6 | 436.9 |  | 5.01 | 4.75 | 6.10 |  | 1.07 | ● | 1.00 |
| *Detonula pumila* | 87.3 | 493.3 | 314.6 |  | ● | ● | 1.03 |  | 1.34 | 1.37 | 1.37 |
| *Guinardia* cf. *delicatula* | 197.0 | 122.0 | 246.2 |  | 2.98 | 1.23 | 1.51 |  | 1.54 | 1.49 | 1.35 |
| *Guinardia* cf. *striata* | 36.1 | 462.5 | 520.3 |  | ● | ● | ● |  | 1.09 | 1.28 | 1.31 |
| *Leptocylindrus danicus* | **617.02** | **7940.6** | **8136.0** |  | 3.36 | 5.54 | 3.77 |  | 2.16 | 2.05 | 2.09 |
| *Meuniera*? | 48.7 | 12.3 | 13.4 |  | 1.66 | ● | ● |  | 1.21 | 1.13 | ● |
| *Odontella mobiliensis* | 7.5 | 4.2 | 10.1 |  | 1.01 | ● | ● |  | ● | ● | ● |
| *Paralia sulcata* | 123.1 | 118.1 | 160.8 |  | 4.91 | 3.3 | 5.24 |  | 1.27 | ● | 1.15 |
| *Pleurosigma* spp. | 6.7 | 4.3 | 11.7 |  | 1.05 | ● | 1.18 |  | ● | ● | ● |
| *Proboscia alata* | 27.4 | 149.8 | 59.0 |  | ● | 1.09 | ● |  | 1.18 | 1.03 | 1.19 |
| *Pseudo-nitzschia* spp. A | 210.6 | 680.7 | 267.1 |  | 1.8 | ● | ● |  | 1.69 | 1.51 | 1.43 |
| *Pseudo-nitzschia* spp. C | 125.8 | **1091.6** | **1816.8** |  | 1.53 | 1.66 | 1.25 |  | 1.65 | 1.65 | 1.68 |
| *Pseudo-nitzschia* spp. D | 6.9 | 635.9 | 975.3 |  | ● | ● | ● |  | ● | ● | 1.12 |
| *Rhizosolenia* cf. *imbricata* | 10.7 | 31.7 | 53.7 |  | ● | ● | 1.08 |  | ● | 1.04 | 1.03 |
| *Thalassionema nitzschioides* | 58.7 | 72.5 | 1165.4 |  | ● | ● | ● |  | ● | 1.10 | 1.10 |
| *Thalassiosira* spp. B | 12.9 | 76.1 | 199.0 |  | ● | ● | ● |  | ● | 1.20 | 1.20 |
| *Thalassiosira* spp. C | 40.0 | 83.3 | **2852.4** |  | 1.13 | ● | 1.89 |  | 1.25 | 1.61 | 1.58 |
| **Dinophyceae** |  |  |  |  |  |  |  |  |  |  |  |
| dinoflagellates A | 47.0 | 90.5 | 74.5 |  | 3.82 | 3.61 | 3.36 |  | ● | ● | ● |
| dinoflagellates B | **414.7** | **963.9** | 893.7 |  | ● | ● | ● |  | 1.60 | 1.51 | 1.38 |
| dinoflagellates naked A | 45.0 | 315.4 | 114.5 |  | ● | ● | 1.02 |  | 1.33 | 1.19 | 1.31 |
| dinoflagellates naked B | 409.0 | 309.6 | 260.1 |  | 1.71 | 2.35 | 1.38 |  | 1.72 | 1.58 | 1.63 |
| dinoflagellates thecate A | 35.6 | 80.0 | 139.2 |  | ● | ● | ● |  | 1.17 | 1.13 | 1.17 |
| *Ceratium furca* | 17.6 | 58.6 | 20.7 |  | 1.06 | ● | ● |  | 1.09 | ● | ● |
| *Ceratium fusus* (300 µm) | 32.0 | 127.9 | 105.7 |  | 1.11 | 1.94 | 1.08 |  | 1.35 | 1.21 | 1.30 |
| *Dinophysis acuta* | 19.7 | 85.9 | 35.2 |  | 1.43 | 2.04 | 1.38 |  | 1.10 | 1.03 | 1.06 |
| *Diplopsalis* sp. | 23.4 | 55.2 | 35.4 |  | ● | 1.16 | ● |  | 1.09 | ● | 1.01 |
| *Gymnodinium* spp. | 5.4 | 38.3 | 74.5 |  | ● | 1.17 | 1.09 |  | 1.03 | ● | 1.07 |
| *Gyrodinium* spp. | 17.6 | 58.6 | 20.7 |  | 1.06 | ● | ● |  | 1.09 | ● | ● |
| *Gyrodinium fusiforme* | 75.2 | 121.5 | 45.4 |  | 2.90 | 3.94 | 2.18 |  | 1.18 | 1.09 | 1.10 |
| *Gyrodinium lacryma* | 7.3 | 23.0 | 28.8 |  | ● | 1.21 | 1.03 |  | ● | ● | ● |
| *Prorocentrum micans* | 17.1 | 29.1 | 11.5 |  | 1.09 | 1.02 | ● |  | 1.07 | ● | ● |
| *Prorocentrum minimum* | 138.1 | **2013.7** | 152.4 |  | 1.00 | 2.45 | 1.11 |  | 1.56 | 1.26 | 1.39 |
| *Protoperidinium bipes* | 13.0 | 14.4 | 14.5 |  | ● | 1.12 | ● |  | ● | ● | ● |
| *Protoperidinium divergens* | 5.8 | 16.2 | 7.9 |  | ● | 1.20 | ● |  | ● | ● | ● |
| *Protoperidinium* spp. | 13.0 | 13.9 | 14.6 |  | ● | 1.00 | ● |  | ● | ● | ● |
| *Scripsiella* cf. *trochoidea* | 162.0 | 563.1 | 446.2 |  | 3.96 | 5.02 | 4.81 |  | 1.34 | 1.20 | 1.16 |
| **Euglenophyceae** |  |  |  |  |  |  |  |  |  |  |  |
| Euglenophyceae und. | 18.9 | 28.62 | 59.7 |  | 1.87 | 1.73 | 2.17 |  | 1.03 | 1.04 | 1.04 |
| *Eutreptiella* spp. | 19.5 | 30.8 | 32.1 |  | ● | ● | 1.01 |  | 1.02 | 1.03 | 1.00 |
| **Haptophyta** |  |  |  |  |  |  |  |  |  |  |  |
| *Emiliania huxleyi* | **2008.5** | **2375.7** | **2329.0** |  | 7.19 | 7.57 | 6.14 |  | 1.57 | 1.52 | 1.42 |
| *Gephyrocapsa* spp. A | **698.0** | **1018.8** | 1245.2 |  | 3.23 | 3.83 | 1.54 |  | 2.06 | 2.00 | 2.00 |
| *Gephyrocapsa* spp. B | 168.1 | 152.3 | 441.9 |  | ● | ● | 1.54 |  | 1.00 | 1.60 | 1.55 |
| *Syracosphaera pulchra* | 29.4 | **1148.1** | 1925.9 |  | ● | ● | ● |  | ● | ● | ● |
|  |  |  |  |  |  |  |  |  |  |  |  |
| % Contribution of selected taxa | 70.0 | 90.4 | 79.4 |  | 56.6 | 62.2 | 57.6 |  | 41.5 | 40.0 | 43.3 |
